# Antisense Versus Antigene in the Computer-Aided Design of Triplex-Forming Oligonucleotides (TFO): Insights from a Dual-Method Review, Combining Bibliometric and Systematic Review

**DOI:** 10.3390/ijms262210936

**Published:** 2025-11-12

**Authors:** Martha Hincapié-López, Jeison Marín-Alfonso, Efrén Romero-Riaño, Rafael Augusto Núñez-Rodríguez, Yarley Vladimir Pabón-Martínez

**Affiliations:** 1 Grupo de Investigación Estudio Genético de Enfermedades Complejas, Universidad Autónoma de Bucaramanga, Bucaramanga 680003, Santander, Colombia; mhincapie162@unab.edu.co; 2 Programa de Ingeniería Electrónica, Unidades Tecnológicas de Santander, Bucaramanga 680005, Santander, Colombia; 3 Department Executive Management, Observatorio Colombiano de Ciencia y Tecnología, OCyT, Bogotá 110231, Cundinamarca, Colombia; eromero@ocyt.org.co; 4 Grupo de Investigación en Control Avanzado—GICAV, Unidades Tecnológicas de Santander, Bucaramanga 680005, Santander, Colombia; rrodriguez@correo.uts.edu.co; 5 Grupo de Investigación Pensamiento Sistémico, Universidad Autónoma de Bucaramanga, Bucaramanga 680003, Santander, Colombia; ypabon561@unab.edu.co; 6 Research Support Office (RSO), External Engagement Office (EEO), Karolinska Institutet, 171 77 Solna, Sweden

**Keywords:** antigene strategy, antisense oligonucleotides (ASO), bibliometric analysis, computer-aided drug design (CADD), in silico modeling, Molecular dynamics simulations, PRISMA, systematic review, triplex forming oligonucleotides (TFO)

## Abstract

This study offers a comprehensive overview of the scientific landscape surrounding computer-aided drug design (CADD) for triplex-forming oligonucleotides (TFOs) within antisense and antigene therapeutic strategies. A dual-method approach was used, combining bibliometric mapping of 6154 Scopus-indexed articles (1980–2023) to identify publication trends and intellectual networks, with a PRISMA 2020-guided systematic review of 62 experimental studies (2015–2024) from Scopus and Web of Science, after removing duplicates using AteneaSIRES. Results show the strong dominance and clinical maturity of antisense strategies, supported by 18 FDA/EMA/MHLW-approved drugs, whereas antigene approaches remain technically limited and underdeveloped. Antigene research has focused on triplex stability modeling and biophysical feasibility but faces challenges with poor biochemical stability, limited in vivo validation, and outdated methods. Meanwhile, antisense design benefits advanced CADD pipelines, including molecular dynamics and docking modeling. Based on these insights, we propose a practical, narrative roadmap as a methodological guide: integrating proven antisense design practices and providing actionable strategies to enhance antigene research, ultimately increasing the translational potential of therapeutic TFOs with solid mechanistic and translational support.

## 1. Introduction

### 1.1. Motivation and Background

Computer-aided methods provide a more accurate way to design, identify, and evaluate drugs compared to traditional approaches. They also enable the discovery and validation of new therapeutic targets, specific molecules, or proteins that a drug is designed to interact with to produce a therapeutic effect.

Computer-aided drug design (CADD) utilizes computational technologies to simulate potential interactions between drugs and their receptors. CADD incorporates in silico analysis at various stages of drug discovery and development, facilitating rapid and easy evaluation before compound synthesis. The use of artificial intelligence (AI) is contributing to the transformation of the drug discovery process in different ways, such as the identification of genes or proteins against diseases [1], the development of high-fidelity molecular simulations [2], the prediction of critical drug properties [3], the generation of promising drug molecules never seen before [1], the ranking and prioritization of lead drug compounds [4], and the generation of synthesis routes for the production of hypothetical drug compounds [1]. All this facilitates the development of new medicines, reducing the time and associated costs, without compromising the quality of the process [5].

Over the past 40 years, the development of oligonucleotides (ONs) has undergone significant improvement [6]. However, only 10 out of 1000 compounds are optimized enough to be evaluated in preclinical trials, a complex approval process that can take up to 15 years [7]. Triplex-forming oligonucleotides (TFOs), a breakthrough in drug development, are synthetic single-stranded DNA molecules that can form triplexes by binding to the major groove in specific regions of the DNA duplex [8], offering a promising future for drug design [9].

Triplex technology is a promising approach [10] for regulating critical biological processes, such as cell signaling, proliferation, and mutagenesis/carcinogenesis [11]. TFOs can regulate transcription, making them molecules with great potential for therapeutic use as ONs [12].

### 1.2. Related Works

**Figure 1:**
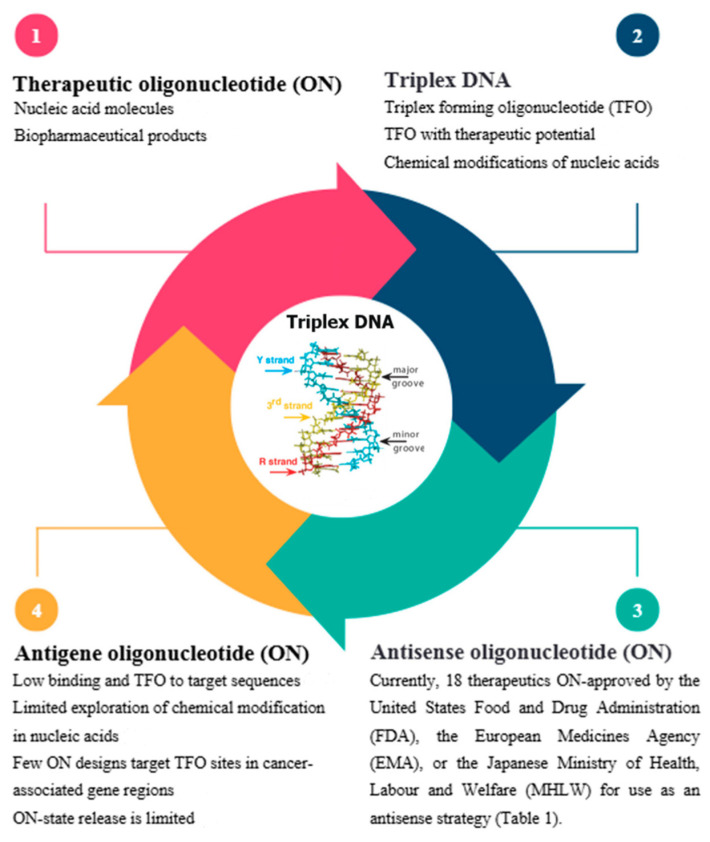
Schematic representation of DNA triplex formation (Source [25]). The core circle shows the target duplex DNA; blue: the pyrimidine strands (Y); red: purine (R) strands; yellow: third strand (TFO) [26]. Section 1. Definition of therapeutic oligonucleotides (ONs) [27,28]. Section 2. Concepts and applications of triplex DNA [9,13,14]. Section 3. Antisense-based oligonucleotides (ONs) that have been approved as therapeutic oligonucleotides (ONs) (Table 1). Section 4. Limitations in the design of antigene-based oligonucleotide (ON) conjugates [29,30,31,32].

Triplex structures in nucleic acids (DNA and RNA) can be formed either between different molecules (intermolecular) or within the same molecule (intramolecular or H-DNA) [13]. The ability of DNA to form intermolecular triplexes has led to the development of TFOs [14], which are designed to bind specifically to the major groove of double-stranded DNA in mammalian genomes [10] and enable targeted gene regulation. TFOs rely on Hoogsteen or reverse Hoogsteen hydrogen bonding [15,16] to form stable triplex structures [14]. TFOs have shown promise as a therapeutic tool [17] with potential for gene regulation [9], particularly in blocking the expression of genes involved in cancer [18], viral infections, and other disorders [19], as well as their ability to interfere with DNA replication [13,20] (Figure 1). This study specifically focuses on the computer-aided design of TFOs, rather than on general TFO biology or therapeutics.

TFOs can be used under two main strategies: as antisense-based ONs [21], where they bind to purine-rich RNA sequences to block translation [22], and as antigene-based ONs [23], where they bind directly to double-stranded DNA to prevent transcription initiation or elongation by occupying regulatory elements [9,12]. Whereas antisense-based ON has achieved notable clinical success, including 18 therapeutics approved by the FDA, EMA, and MHLW (Table 1; Figure 1 and Section 3), antigene strategies remain significantly underexplored, highlighting the importance of our research.

Although TFOs were originally developed as antigene agents, capable of regulating gene expression through triple helix formation [24], their use has increasingly overlapped with antisense-based ON strategies, especially when sequence complementarity allows RNA targeting without triplex formation [25]. Notably, while the antisense field has adopted computer-aided design tools to optimize binding affinity, stability, and specificity, antigene-directed TFOs have lagged, often limited by sequence constraints and biophysical instability. This divergence is reflected in therapeutic outcomes: Antisense-based ONs have led to 18 FDA, EMA, and MHLW-approved drugs to date. Conversely, there is no clinically approved antigene-based TFO (Figure 1, Section 4). This review examined how computational design has contributed to this disparity, assessing the scientific landscape of TFO design in both antisense and antigene strategies.

**Table 1:** Therapeutic oligonucleotides (ONs) approved by the FDA, EMA, or MHLW using the antisense strategy.

On Drug	On Class	Approval Date	Indication	Target Gene (AR)	Developing Company	Ref
Tofersen (Qalsody™)	siRNA	2023 *	Amyotrophic lateral sclerosis (ALS)	*SOD1* CNS (IT)	Ionis Pharma/ Biogen	[21]
Volanesorsen (Waylivra^®^)	siRNA	2019 **	Familial chylomicronemia syndrome	*ApoC-III* Liver (SC)	Ionis Pharma/ Akcea Tx	[33]
Inotersen (Tegsedi^®^)	Gapmer PS-ASO	2018 * 2018 **	Hereditary transthyretin amyloidosis	*hTERT* Liver (SC)	Ionis Pharma/ Akcea Tx	[34]
Mipomersen (Kynamro^®^)	Gamper PS-ASO	2013 *	Homozygous familial hypercholesterolemia (HoFH)	*ApoB*-100 Liver (SC)	Ionis Pharma/Genzyme/Kastle Tx	[35]
Lumasiran (Oxlumo^®^)	siRNA/ GalNAc conjugate	2020 *	Primary hyperoxaluria type 1	*HAO1* Liver (SC)	Alnylam Pharma	[36]
Inclisiran (Leqvio^®^)	siRNA/ GalNAc conjugate	2020 **	Hypercholesterolemia or mixed dyslipidemia	*PCSK9* Liver (SC)	Alnylam Pharma /Medicines/ Novartis	[37]
Givosiran (Givlaari^®^)	siRNA/ GalNAc conjugate	2019 *	Acute hepatic porphyria	*ALAS*1 Liver (SC)	Alnylam Pharma	[38]
Patisiran (Onpattro^®^)	LNP-siRNA	2018 *	Transthyretin amyloidosis	*hATTR* Liver (IV)	Alnylam Pharma	[39]
Defibrotide (Defitelio^®^)	ssDNA/aptamer	2016 *	VOD in HSCT	*VOD/SOS* Liver (IV)	Jazz Pharma	[40]
Vutrisiran (Amvuttra^TM^)	siRNA	2022 *	Transthyretin amyloidosis	*wtATTR* Heart (SC)	Alnylam Pharma	[41]
Casimersen (Amondys 45^®^)	PMO ASO	2021 *	Duchenne muscular dystrophy (DMD)	*Dystrophin* exon 45 Muscle (IV)	Sarepta Tx	[42]
Viltolarsen (Viltepso 53^®^)	PMO ASO	2020 ** 2020 ***	Duchenne muscular dystrophy	*Dystrophin* exon 53 Muscle (IV)	Nippon Shinyaku Pharma	[43]
Golodirsen (Vyondys 53^®^)	PMO ASO	2019 *	Duchenne muscular dystrophy	*Dystrophin* exon 53 Muscle (IV)	Sarepta Tx	[44]
Milasen (N-of-1 ASO)	Splice- switch ASO	2018 *	Mila Makovec CLN7 gene associated with Batten disease	*MFSD8* CNS (IT)	Boston Children’s Hospital	[45]
Nusinersen (Spinraza^®^)	Splice- switch ASO	2016 * 2017 **	Spinal muscular atrophy	*SMN*2 intron 7 CNS (IT)	Ionis Pharma/ Biogen	[46]
Eteplirsen (Exondys 51^®^)	PMO ASO	2016 *	Duchenne muscular dystrophy	*Dystrophin exon 51* Muscle (IV)	SareptanTx	[47]
Pegaptanib (Macugen^®^)	Aptamer	2004 *	Macular degeneration	*VEGFA* Eye (ITV)	NeXstar Pharma/Eyetech Pharma	[48]
Fomivirsen (Vitravene^®^)	ASO	1998 * 1999 **	Cytomegalovirus retinitis	*CMV IE*2 Eye (ITV)	Ionis Pharma/Novartis	[49]

Mechanism of action for ON class: RNase H-mediated degradation of mRNA (shaded in pale lilac); RISC-mediated degradation of mRNA (shaded in pale grey); splice-switching (shaded in pale pink); translation block (shaded in pale blue). Abbreviations: ON (oligonucleotide); FDA (United States Food and Drug Administration); EMA (European Medicines Agency); MHLW (Japanese Ministry of Health, Labour and Welfare); AR (administration route); siRNA (small interfering RNA); *SOD1* (superoxide dismutase 1; CNS (central nervous system); IT (intrathecal); *ApoC3* (apolipoprotein C-III); SC (subcutaneous); PS (phosphorothioate); ASO (antisense oligonucleotide); *hTERT* (Telomerase Reverse Transcriptase); *ApoB* (apolipoprotein B); GalNac (N-Acetyl galactosamine); *HAO1* (human 2-hydroxy acid oxidase 1); *PCSK9* (proprotein convertase 9); *ALAS*1 (5′-aminolevulinate synthase 1); LNP (lipid nanoparticle); *hATTR* (hereditary transthyretin-mediated); IV (intravenous); ssDNA (single strand DNA); *VOD* (veno-occlusive disease), *HSCT* (human stem cell transplantation); *SOS* (sinusoidal obstruction syndrome); *wtATTR* (amyloid deposits contain variant and wild-type of transthyretin-mediated); PMO (phosphorodiamidate morpholino oligomer); *MFSD8* (main domain of the facilitator superfamily containing 8); *SMN2* (survival of motor neuron 2, telomeric); *VEGFA* (vascular endothelial growth factor A); ITV (intravitreal); *CMV* (cytomegalovirus); *IE*2 (immediate early protein 2); * approved by the FDA; ** approved by the EMA; *** approved by the MHLW.

### 1.3. Contributions

This study offers an overview of computer-aided TFO design in antisense and antigene therapeutic strategies. The main contributions are as follows:

1. Highlighting a clear imbalance: while antisense ONs have achieved significant clinical maturity, antigene approaches remain underexplored, revealing a persistent technological gap.
2. To address this gap, we suggest incorporating advanced computational methods to overcome current challenges in TFO design and improve their therapeutic potential.

### 1.4. Manuscript Research Questions and Organization

Despite the conceptual appeal of TFOs—particularly their potential to suppress gene expression at the DNA level—antigene strategies have not yet achieved translational success due to persistent biochemical and technical challenges [50] (Figure 1 and Section 4). These include the requirement for homopurine–homopyrimidine stretches as triplex target sites (TTSs) [51], which are not ubiquitously present in gene promoters; low thermal and biological stability of triplex structures [52]; pH sensitivity [53]; electrostatic repulsion between DNA strands [52]; and poor cellular delivery and bioavailability [29]. Moreover, unmodified TFOs are prone to rapid degradation, often exhibiting short half-lives in biological systems [54].

These limitations have shifted research efforts toward CAAD of TFO. This change has not only highlighted the challenges but also the significant progress made in ASO research. The multiple FDA-, EMA-, and MHLW-approved therapies using ASOs demonstrate this progress (Table 1). However, it is important to note that antigene-targeting TFOs, although underdeveloped, still hold potential (Figure 1, Section 1 and Section 4).

Given this context, the present review addresses the following overarching research question. This question is important within the field of nucleic acid therapeutics and ON design. It has the potential to guide future research and influence the direction of the field. To guide this investigation, we formulated the following overarching research question.

How have the research landscape and experimental applications of TFOs evolved in antisense and antigene strategies, and what methodological and experimental gaps hinder the progress of antigene approaches?

To address this, we employed a dual methodological approach, combining both quantitative and qualitative methods. This approach was selected to provide a comprehensive understanding of the research landscape and experimental use of TFO, focusing on two specific areas of sub-questions:

- Bibliometric question: What are the publication trends, thematic clusters, and intellectual networks related to the design and application of TFO in antisense and antigene contexts?
- Systematic review question (PRISMA): What experimental criteria and design features have been reported for TFO in antigene strategies, and how do these compare to those used in antisense applications?

**Figure 2:**
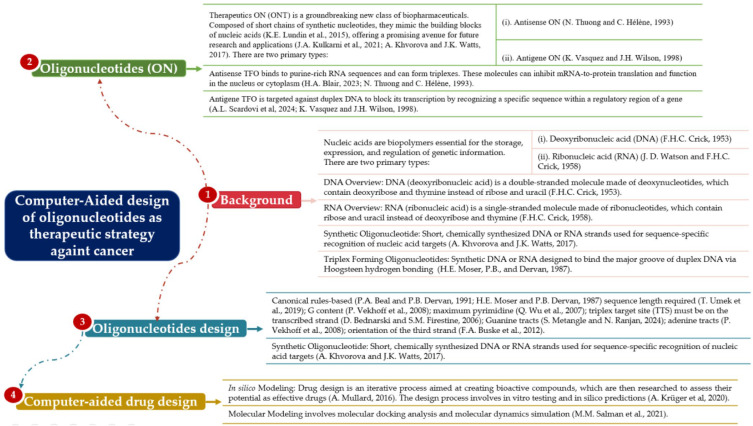
Conceptual map of the theoretical framework. The framework situates computer-aided oligonucleotide (ON) design as a therapeutic strategy against cancer, encompassing nucleic acid structures [12,21,28,56,57,58,59], therapeutic oligonucleotide (ONT) definitions [28,60,61,62], triplex-forming oligonucleotide (TFO) design rules [10,28,62,63,64,65,66,67,68], and computer-aided drug design (CADD) principles [69,70].

This review is divided into four main sections:

Section 1, the Introduction, provides motivation and background. CADD enhances accuracy in target identification and molecular simulation, decreasing development time and costs. By integrating AI, CADD speeds up drug discovery, including the rational design of new compounds. Section 2. Conceptual and Theoretical Framework: Provides background on nucleic acid structures, the classification and function of synthetic ONs, the molecular basis of triplex formation, and principles of computer-aided design (see Figure 2). Section 3. Methods: Describes the bibliometric methodology based on the Scopus-listed literature from 1980 to 2023 and the PRISMA 2020-compliant systematic review using Scopus (2015–2025) and Web of Science (2015–2025), detailing search strategies, filtering criteria, de-duplication software (AteneaSIRES v1.0.3, https://ateneasires.com/) [55], and quality control with R/Bibliometrix. Section 4. Results and Discussion: This section presents key findings from both analytical streams, compares antisense and antigene strategies, identifies experimental gaps, and outlines canonical design rules for TFO. It synthesizes bibliometric and PRISMA findings, highlighting the imbalance between antisense and antigene strategies, and outlines the study’s novel methodological and conceptual contributions. Section 5. Conclusions: Summarizes the clinical and technical implications, highlights the ongoing gap in antigene TFO development, and suggests computational pipelines as a roadmap for future research.

This combined bibliometric and systematic analysis offers both a broad overview of scientific output and detailed insights into experimental methods. By mapping the structure and progress of TFO research, this study helps clarify why antigene strategies remain rare and how computational design could support reinvigorating their therapeutic potential.

### 1.5. Conceptual and Theoretical Framework

The following subsections outline the conceptual and theoretical foundations that support this study, ranging from nucleic acid structures to principles of computer-aided design.

#### 1.5.1. Nucleic Acids’ Structures

Nucleic acids are essential biopolymers that store, express, and regulate genetic information. The main types include deoxyribonucleic acid (DNA) and ribonucleic acid (RNA). DNA is typically a double-stranded helix made up of deoxyribonucleotides and serves as the long-term storage of genetic information in most organisms [56]. RNA, generally single-stranded and composed of ribonucleotides, performs diverse roles, including transcription, translation, catalysis, and gene regulation [57]. Both molecules rely on base pairing and structural folding to perform their biological functions [58] (Figure 2). DNA mainly adopts the B-form helix with Watson–Crick base pairs (A:T, G:C); triplex formations occur through Hoogsteen base pairing (A:T·A, G:C·+ G). DNA can also form alternative conformations [7], including Z-DNA [59], fork and triplex structures [60], and G-quadruplexes [61]. RNA with Watson–Crick base pairing can also form non-canonical pairs in secondary structures and folds into complex secondary and tertiary formations, such as hairpins, loops, and pseudoknots, which are critical for its functional versatility [62]. These structural motifs can also hinder hybridization efficiency, as regions engaged in hairpins, loops, or pseudoknots become partially inaccessible to complementary binding by antigene TFOs. Such steric and topological constraints reduce the efficiency of triplex formation and complicate the prediction of Hoogsteen-compatible sites. While antisense-oriented CADD tools (e.g., from NUPACK (2010) [63] to SNUPI (2021 [64]) implement thermodynamic algorithms to estimate local accessibility and unfolding energy [65], no equivalent optimization framework yet exists for antigene TFOs targeting structured RNA, representing an unmet challenge in current triplex modeling. Additionally, certain types of RNA, like small interfering RNA (siRNA), microRNA (miRNA), aptamers, riboswitches, and antisense RNAs, can be artificially engineered or modulated for therapeutic and biotechnological applications. This engineering leverages their ability to bind with target molecules through base complementarity, opening new avenues for RNA in the field of therapeutics [66]. The field of molecular biology and bioinformatics has been revolutionized by advances in technology, enabling the design of synthetic ONs derived from both DNA and RNA for therapeutic and biotechnological applications [28].

#### 1.5.2. Synthetic Oligonucleotides

Synthetic ONs are short (usually 13–25 nt long) chemically synthesized DNA or RNA strands designed to hybridize with complementary nucleic acid sequences. This broad category includes both ASOs, which target messenger RNA (mRNA) [58], and the TFOs, which bind the major groove of double-stranded DNA to form a triple helix and modulate transcription [14] (Figure 2).

**Figure 3:**
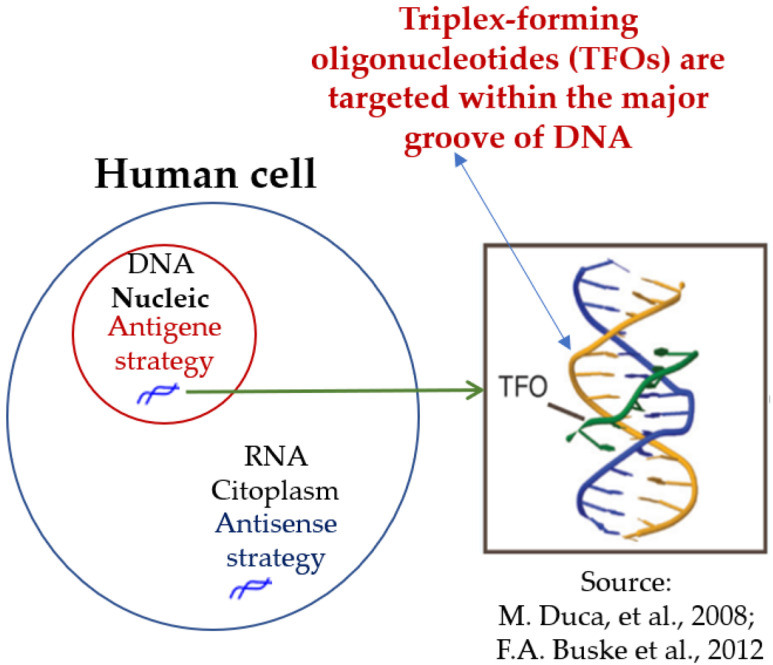
Comparative target accessibility of antisense and antigene strategies. These schematic contrasts antisense oligonucleotides (ASO), which act in the cytoplasm by binding accessible single-stranded mRNA to silence gene expression, with triplex-forming oligonucleotides (TFOs), which must enter the nucleus and recognize homopurine–homopyrimidine tracts in duplex DNA via Hoogsteen hydrogen bonds. The box shows a triplex structure. Limited DNA accessibility in vivo, due to chromatin compaction, pH dependence, and electrostatic repulsion, remains a major barrier to antigene translation [26,66].

Synthetic ONs characterize both ASOs and TFOs, yet ASOs dominate the literature and clinical application for several reasons: (i) Clinical maturity: Over 18 ASO-based therapeutics have been approved by the FDA, EMA, and MHLW for conditions ranging from spinal muscular atrophy and Duchenne muscular dystrophy to amyotrophic lateral sclerosis (e.g., nusinersen [46], eteplirsen [47], inotersen [34], viltolarsen [43], to name a few). (ii) Extensive chemical and pharmacokinetic characterization: ASOs have been extensively investigated for backbone modifications (e.g., phosphorothioate [46]), sugar modifications (e.g., 2′-MOE [33], LNA [9]), and delivery conjugates [37]. These modifications enhance nuclease resistance, binding affinity, and biodistribution, as highlighted in comprehensive reviews [67]. (iii) Target accessibility: The ASO acts on RNA, being single-stranded and often localized in the cytoplasm, making it more accessible for hybridization than genomic DNA embedded in chromatin [68]. Targeting nuclear DNA with TFOs remains less feasible in vivo [69] due to the limited translation of antigene approaches [70] (Figure 3).

Despite their conceptual appeal and sequence-specific ability to regulate gene expression at the transcriptional level, TFOs remain largely preclinical [12], facing challenges such as sequence constraints [51], pH and thermal sensitivity [53], and delivery barriers [30].

#### 1.5.3. Triplex-Forming Oligonucleotides (TFOs)

TFOs are synthetic DNA or RNA strands designed to bind the major groove of double-stranded DNA via Hoogsteen hydrogen bonding [15,16] (Figure 2). These molecules exploit the intrinsic ability of nucleic acids to form triplex structures, either intermolecular or intramolecular, such as in H-DNA [13,24]. The triplex target site (TTS) is a homopurine–homopyrimidine stretch within the DNA duplex that accommodates the TFO for specific binding to the major groove [10,71]. Notably, promoter regions are enriched in TTS motifs, making them preferred loci for triplex formation [72]. This unique ability of the TFO to interfere with the transcriptional machinery by obstructing the RNA polymerase-DNA-DNA complex, thus providing a mechanism for gene expression control [73], is a fascinating area of study that continues to intrigue researchers and professionals in the field.

Given these unique properties, TFOs hold significant potential in the rapidly advancing field of synthetic biology. This discipline applies engineering principles to biological systems to create new functionalities, optimize existing pathways, and address practical challenges in medicine and biotechnology [74]. Recent advances in synthetic biology include automated sequence design, genetic component standardization, and modular DNA synthesis and assembly, all of which may enhance the rational development and application of TFOs [75]. The potential of TFOs in synthetic biology is extensive, and their unique binding mechanism to double-stranded DNA makes them a promising tool for future research and applications [17].

#### 1.5.4. Therapeutic Oligonucleotides (ONTs)

ONTs are a new class of biopharmaceuticals comprising short, chemically modified nucleotide chains that mimic natural nucleic acids, enabling precise gene regulation [76] (Figure 2). These molecules can target a wide range of tissues but often face biological barriers, requiring specialized delivery methods. ONTs are given systemically, mainly subcutaneously or intravenously, and through localized techniques such as intrathecal, intravitreal, ocular, intradermal, intratumoral, mucosal, oral, or inhalation delivery. To enhance systemic stability and tissue targeting, they are sometimes conjugated to ligands (e.g., GalNAc) or incorporated into delivery vehicles, such as nanoparticles [77].

ONTs currently approved by the FDA, EMA, and MHLW use several mechanisms: (i) *Antisense oligonucleotides* (ASOs), which trigger RNase H-mediated mRNA degradation [49]. (ii) *Splice-switching oligonucleotides* (SSOs) that modulate pre-mRNA splicing [46]. (iii) *Small interfering RNA* (siRNA) that directs RNA-induced silencing complexes (RISCs) [33]. (iv) *Peptide nucleic acids* (PNAs) and phosphorodiamidate morpholino oligomers (PMOs) [43]. (v) *siRNA conjugated with GalNAc* for efficient liver targeting [37]. (vi) *siRNA encapsulated in lipid nanoparticles* (LNPs) for systemic delivery [39].

A detailed overview of the FDA-, EMA-, and MHLW-approved ONTs is provided in Table 1. This table includes key details such as the approval year, regulatory agency, molecular target, associated disease, pharmaceutical developer, and bibliographic reference. It emphasizes the current clinical uses of ONTs, including the mechanisms that explain the growing diversity and increasing maturity of ON-based therapeutics [6], fostering confidence in the progress of this field.

These advances demonstrate the therapeutic versatility of ONTs across different delivery methods and molecular targets. While they show potential for antigen therapy, TFOs have not yet received regulatory approval and remain in the preclinical research stage.

#### 1.5.5. Antisense and Antigene Oligonucleotides

TFOs have been proposed as potential therapeutic tools because of their demonstrated ability to inhibit the expression of genes involved in cancer development [12] and other human diseases [19]. They can also interfere with DNA replication by forming stable triple helix structures through sequence-specific base recognition [13,20]. TFOs are chemically synthesized DNA or RNA strands that can bind to the major groove of duplex DNA at triplex target sites (TTSs) [51], which are specific sequences within the DNA that are particularly rich in either purines or pyrimidines, allowing for stable triple helix formation [14]. These interactions can be used to modulate gene expression through an antisense or antigenic strategy.

Antisense Oligonucleotide Strategy

In the antisense approach, TFOs are designed to hybridize with specific RNA sequences, often rich in purines, forming ON duplexes that block gene expression post-transcriptionally (Figure 2). This can occur either in the nucleus or cytoplasm [26]. Antisense-based TFOs primarily act through three mechanisms:

- RNase H-mediated degradation: When hybridized to target mRNA, the antisense-based TFO recruits RNase H, an endogenous endonuclease that degrades the RNA strand of DNA–RNA hybrids [78]. It then leaves the ASO intact, allowing for sustained inhibition [67]. The recognition efficiency depends on the TFO’s chemical modifications [28].
- Steric blockade of translation: Antisense-based TFOs can bind to essential mRNA regions like the 5′ cap, ribosome binding site, or start codon, physically blocking ribosome assembly and thereby inhibiting translation [78]. This steric interference also affects alternative splicing and causes mRNA destabilization in the nucleus [79].
- Splice-switching and transcript processing inhibition: Splice-switching ONs (SSOs) can regulate pre-mRNA splicing by preventing the binding of spliceosomal components, leading to exon skipping or intron retention [80]. Antisense-based TFOs can also block polyadenylation and transcript maturation by targeting related signals [81].

Antigene Oligonucleotide Strategy

**Figure 4:**
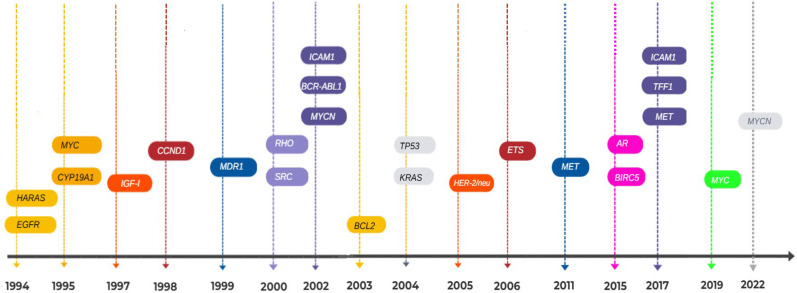
Timeline of genes targeted by antigene oligonucleotides (ONs). Chronological overview (1994–2022) of oncogenes and regulatory genes targeted by triplex-forming oligonucleotides (TFOs) through an antigene approach. The timeline shows initial interest, a variety of molecular targets, and gradual progress toward clinical use. However, no antigene-based TFO has yet received regulatory approval, highlighting ongoing difficulties with design optimization, delivery, and validation.

Conversely, the antigene strategy uses TFOs that directly bind to duplex DNA at regulatory promoter or enhancer regions, thereby blocking transcription initiation [8]. These antigene TFOs form triple helices within the DNA major groove, often requiring a TTS of homopurine–homopyrimidine sequences [51] (Figure 2). Numerous antigene-based TFOs have been designed against oncogenes and transcription factors such as *c-MYC* [82], *BCL2* [83], *N-MYC* [12], *EGFR* [84], *p53* [85], and *Ki-RAS* [86], among others (Figure 4). Despite this, no antigene-based TFO has yet been approved for clinical use, mainly due to biophysical limitations such as low intracellular stability [52], pH sensitivity [53], electrostatic repulsion [52], and poor nuclear delivery [30]. Ongoing research on chemical modifications, conjugation techniques, and delivery systems is essential for stabilizing antigene TFOs and enhancing their sequence specificity while reducing cellular toxicity.

#### 1.5.6. Oligonucleotide Design

The practical design of a TFO requires a careful balance of thermodynamic, structural, and biochemical parameters. This balance is crucial for high binding affinity, sequence specificity, and biological stability [52]. A critical aspect is the efficient recognition of unique TTSs [51]. Key factors to consider include physiological pH, ionic strength [53], and minimal degradation by endonucleases [87]. Guided by canonical triplex formation rules [88,89], one could apply rigorous selection parameters to optimize the performance of the antigene TFO (Figure 2).

Triplex structures are formed through Hoogsteen or reverse Hoogsteen hydrogen bonding, depending on the orientation of the third strand relative to the purine-rich strand of the duplex [69]. These interactions form stable triplets, arranged reliably in trinucleotide triads (e.g., T·A–T or C+G–C), where the TFO recognizes and binds to a homopurine–homopyrimidine stretch within duplex DNA [54].

Canonical Design Rules

- Minimum TTS Length. The length of the TTS is a key factor that directly affects TFO specificity [51]. It is essential to note that short TTS sequences (e.g., 15 nucleotides) have a less than 1% chance of being unique in the genome [23]. However, the length of ≥21 nt significantly increases uniqueness to over 50% [90], while a TTS ≥ 24 nt achieves approximately 90%, and those ≥ 26 nt are virtually unique [10]. Therefore, longer TTSs are preferred to reduce off-target interactions [51], a key insight for your research on DNA targeting and gene regulation [12].
- Maximum Pyrimidine Interruptions. Then, it is essential to understand that TFOs bind to homopurine DNA strands through Hoogsteen base pairing. Interruptions by pyrimidines within the TTSs markedly decrease triplex stability and binding affinity due to energetic penalties from mismatches [91]. For optimal specificity and triplex stability, it is advisable to limit the number of pyrimidine interruptions to one [10]. This cautionary note should guide your selection process for TTS.
- Location of TTSs on the Transcribed Strand. Triplex formation can suppress gene expression either at the promoter, blocking transcription initiation, or within the coding region, hindering elongation. Although targeting the promoter is conceptually attractive, TFOs often encounter access limitations due to bound transcription factors [92]. In contrast, the transcribed region offers more accessible TTSs and fewer steric hindrances [93]. Therefore, TFOs should ideally target the transcribed strand within coding regions, employing genome-wide alignment to ensure site uniqueness and reduce off-target effects [17,51].
- Guanine Tracts. TTS sequences containing long guanine tracts (>3 G) may form G-quadruplex structures, which compete with triplex formation and decrease binding efficiency [19,94]. Therefore, it is essential to prevent sequences that could form such secondary structures during TTS selection [95]. This awareness will assist your TTS targeting and gene regulation research [51].
- Adenine Tracts. Adenine content of no more than seven nucleotides [95] in a sequence segment.

Non-Canonical Design Rules

Beyond traditional sequence rules, the effectiveness of a TFO is significantly influenced by biochemical and structural factors, including high target-binding affinity, physiological pH, and ionic strength [96], resistance to nucleases, and strong sequence specificity [6,19]. These parameters are frequently enhanced by adding chemical modifications to the nucleic acid structure (Figure 2).

Such modifications can target three main regions of the nucleotide: the nucleobase, the sugar moiety, and the phosphodiester bond.

- Base modifications, such as methylation or the incorporation of unnatural bases, can improve binding affinity to purine-rich sequences and enhance Hoogsteen pairing essential for triplex formation for DNA [88] and for RNA [97].
- Sugar modifications, such as locked ribose conformations (e.g., in LNA) or conformational constraints (like in tricyclo-DNA), enhance triplex thermal stability and nuclease resistance [73].
- Phosphodiester backbone modifications, such as phosphorothioate or phosphoramidate linkages, decrease nuclease susceptibility and change charge distribution, enhance biostability and cellular uptake for DNA [12,98], and for RNA [99].

Key synthetic ONs chemistries used in TFOs include phosphorodiamidate morpholino oligomers (PMO), which possess a neutral backbone resistant to enzymatic degradation [42]; tricyclo-DNA (tcDNA), which enhances affinity and cellular uptake [100]; peptide nucleic acids (PNAs), which replace the sugar–phosphate backbone with a peptide-like structure that increases hybridization strength and biostability [12]; and locked nucleic acids (LNAs), which feature a methylene bridge locking the ribose ring in a favorable conformation, enhancing both thermal stability and mismatch discrimination [23,101]. Each of these offers specific advantages, including increased binding strength, enhanced mismatch discrimination, reduced off-target effects [17], and improved pharmacokinetic properties [23,79,96].

These rational modifications are crucial for addressing key challenges in antigene strategies, such as low in vivo stability, electrostatic repulsion, and chromatin inaccessibility, thereby supporting the development of next-generation TFOs for clinical use [17].

#### 1.5.7. Computer-Aided Drug Design (CADD)

The power of CADD has transformed the landscape of therapeutic molecule development. This innovative method, which utilizes computational tools to model and predict how molecules interact with biological targets, has accelerated drug discovery. By allowing early-stage in silico assessments, CADD has significantly reduced the time, cost, and failure rates typically associated with experimental screening, ushering in a new era of drug discovery [1,5].

CADD plays a crucial role in designing TFOs, which target the major groove of double-stranded DNA. It is essential for evaluating the stability, binding affinity, and biological specificity of TFOs before synthesis. The integration of CADD with genomics, cheminformatics, and artificial intelligence enables the rational design of antigene TFOs with improved therapeutic potential [1,2].

In Silico Modeling

The drug development process provides an efficient and cost-effective way to simulate, predict, and optimize the behavior of candidate molecules (Figure 2). TFOs are designed to bind specific polypurine-rich sequences within genomic DNA—known as a TTS—in the promoter or coding regions for antigene strategies [8,89].

These methods enable the prediction of physicochemical properties and pharmacokinetic behavior using absorption, distribution, metabolism, excretion, and toxicity (ADMET) models [3]. Quantitative Structure–Activity Relationships (QSARs) and Quantitative Structure–Property Relationships (QSPRs) connect molecular structure to function, enabling the identification of high-affinity TFO candidates with desirable therapeutic profiles [102].

Advanced machine learning (ML) algorithms, including both supervised and unsupervised models, improve the interpretation of high-dimensional biological data and facilitate the prioritization of candidate sequences [103]. These models are improved through hyperparameter optimization to ensure reliable predictions in TFO sequence activity and selectivity.

Molecular Modeling

Molecular modeling tools enhance TFO design by simulating the spatial and energetic aspects of ligand–target interactions.

- Molecular docking predicts the binding conformation and affinity of a TFO within the major groove of the duplex DNA target. Depending on the structural flexibility, docking protocols are classified as rigid–rigid, flexible–rigid, or flexible–flexible [1]. Scoring functions and ligand sampling algorithms are designed to distinguish between productive and non-productive interactions by evaluating the steric and electrostatic complementarity between TFOs and target DNA. TFO candidates are also evaluated for potential off-target binding [17,51] through homology screening [104] against genomic databases.
- Molecular dynamics simulations (MDS) are essential for gaining atomistic insights into the stability of TFO–DNA triplexes over time under physiological conditions [31]. These simulations model conformational changes, hydrogen bonding, and water-mediated interactions, providing a comprehensive understanding of the key factors that contribute to triplex stability and specificity. This detailed approach to research using MDS should reassure the audience about the reliability of the TFO design [2,105].

## 2. Methods

We used a dual-method approach to analyze the scientific and experimental landscape of computer-aided TFO design. First, a bibliometric analysis was conducted to map publication trends, research networks, and thematic structures. A systematic review was conducted in accordance with the PRISMA 2020 guidelines [106] to synthesize experimental evidence and methodological criteria.

### 2.1. Database Selection and Bibliometric Evaluation Criteria

To ensure the inclusion of the scientifically rigorous and peer-reviewed literature, this review was restricted to two internationally recognized bibliographic databases: Scopus (Elsevier) [107] and Web of Science Core Collection (Clarivate Analytics) [108]. These platforms were selected due to their broad multidisciplinary coverage in biomedical, chemical, and computational sciences, as well as their strict indexing standards and advanced filtering options based on publication type, subject area, language, and impact metrics.

Both databases provide access to structured metadata exports and verified journal-level metrics, facilitating selective literature screening. Specifically, the review used the following bibliometric indicators (Table 2):

- From Scopus: CiteScore, SJR (SCImago Journal Rank), and SNIP (Source-Normalized Impact per Paper) [109].
- From Web of Science: Impact Factor (IF), Journal Citation Indicator (JCI), and Eigenfactor Score [110].

To ensure high-impact and relevant sources, only articles published in Q1 or Q2 journals, or those with a CiteScore of 3 or higher or an Impact Factor of 3 or higher, were included in the final analysis. Table 2 summarizes the key features of both databases, including their filtering capabilities, bibliometric tools, and their utility in identifying literature on the computational design of TFOs. By relevance, we mean the database’s ability to offer comprehensive coverage of TFO-related research and its tools for bibliometric analysis in this specific area.

Scopus [107] was used for both the bibliometric analysis and the PRISMA-based systematic review [106] as it offers advanced citation mapping, journal-level metrics, and exportable structured data ideal for science mapping and performance analysis. In contrast, Web of Science [108] was used solely for systematic review due to its curated indexing and compatibility with PRISMA-compliant literature selection protocols. Including both databases enhanced the reliability and comprehensiveness of the evidence base, while reducing bias due to database-specific coverage limitations.

### 2.2. Search Strategy or Eligibility Criteria

A structured Boolean search was developed to identify studies focused on the computational design of TFOs in the context of antisense and antigene therapeutic strategies. The query for bibliometric analysis (Box 1) combines three conceptual categories: (i) keywords defining the molecular entities of interest (e.g., TFOs, ASOs, RNA); (ii) computational methodologies (e.g., in silico modeling, MD, computer-aided design); and (iii) their therapeutic context (e.g., antisense or antigene strategies). To improve specificity, we excluded peer-reviewed articles related to G-quadruplex structures, which are topologically distinct and not involved in triplex formation. This search strategy was designed to maximize thematic relevance and minimize conceptual overlap with unrelated ON architectures.

Box 1 **Search equations for bibliometric analysis**

TITLE-ABS-KEY ( ( ( “adenine” OR “base sequence” OR “base pair” OR “base pairing” OR “binding affinity” OR “binds duplex” OR “binding site” OR “cancer” OR “chemical modification” OR “clinical trial” OR “cytosine” OR “chemical structure” OR “DNA” OR “DNA conformation” OR “deoxyribonucleic acid” OR “dsDNA” OR “DNA binding” OR “drug delivery systems” OR “drug approval” OR “DNA sequence” OR “drug therapy” OR “DNA duplex” OR “duplex DNA” OR “duplex” OR “DNA sequence” OR “double-stranded” OR “double-stranded DNA” OR “DNA triplex” OR “DNA helix” OR “enhancer” OR “enhancer element” OR “genetic engineering” OR “FDA” OR “enhancer-binding” OR “gene” OR “gene editing” OR “gene expression” OR “gene silencing” OR “gene transcription” OR “genetic transcription” OR “gene therapy” OR “genetic therapy” OR “gene expression regulation” OR “genetic disease” OR “guanine” OR “G-rich triplexes” OR “genome” OR “gene targeting” OR “genetic transcription” OR “hairpin” OR “high-affinity binding” OR “homoduplex” OR “Hoogsteen” OR “Hoogsteen interaction” OR “Hoogsteen hydrogen bond” OR “Hoogsteen base pairing” OR “hybridization” OR “homologous recombination” OR “H-DNA” OR “*H-DNA” OR “hydrogen bond” OR “hydrogen bonding” OR “homopurine” OR “hydrogen bonding interaction” OR “polypurine sequence” OR “purine” OR “purine-rich strand” OR “purine stretch” OR “homopurine-homopyrimidine” OR “purine-pyrimidine” OR “homopyrimidine” OR “pyrimidine” OR “homopyrimidine-homopurine” OR “pyrimidine-purine” OR “homopyrimidine-pyrimidine” OR “intracellular stability” OR “ionic strength” OR “infection disease” OR “intermolecular bonds” OR “intermolecular triplex” OR “intramolecular bonds” OR “intramolecular triplex” OR “intramolecular triplex-helix” OR “major groove” OR “non-B DNA” OR “nucleotide” OR “Nervous System Disease” OR “NCBI” OR “nucleic acid” OR “nucleic acid conformation” OR “nucleotide sequence” OR “nuclear localization signal” OR “oligodeoxyribonucleotide” OR “oligodeoxynucleotide” OR “oligomer” OR “off-target effects” OR “oligonucleotide design” OR “oligonucleotide” OR “RNA” OR “ribonucleic acid” OR “oligonucleotide-based therapeutics” OR “Oligonucleotide Based Therapy” OR “physiological conditions” OR “precision medicine” OR “personalized medicine” OR “polymerase II” OR “promoter region” OR “genetic promoters” OR “promoter” OR “reverse Hoogsteen” OR “replication” OR “recombination” OR “small RNA” OR “RNA therapeutics” OR “Rnase H” OR “small non-coding RNA” OR “messenger RNA” OR “small interfering RNA” OR “RNA-induced silencing complex” OR “RNA polymerase” OR “RNA polymerase II” OR “microRNA” OR “RNA interference” OR “RNAi therapeutic” OR “small-molecule” OR “single strand” OR “single-stranded DNA” OR “supercoiled DNA” OR “superhelical DNA” OR “screening” OR “synthetic DNA” OR “specific sequence” OR “sequence-specific DNA-binding” OR “therapeutics” OR “therapy” OR “thymine” OR “TATA box” OR “transcription” OR “transcription initiation site” OR “transcription start site” OR “transcription factor” OR “transcription elongation” OR “United States Food and Drug Administration” OR “Watson-Crick” OR “Watson-Crick base pairing” OR “Watson-Crick hydrogen bond” OR “Watson-Crick interaction”)) AND (“antisense” OR “antisense strategy” OR “antisense oligonucleotide” OR “antigene” OR “antigene strategy” OR “antigene oligonucleotide” OR “antiparallel” OR “antiparallel triplex helix” OR “B-DNA” OR “protonated triplex DNA” OR “triple-stranded” OR “triplex intercalator” OR “triplex-forming motifs” OR “triplex-forming sequence” OR “Triplex-Forming Oligonucleotides” OR “triplex forming oligonucleotide” OR “triplex-forming oligonucleotide” OR “triplex-forming oligonucleotides” OR “TFO” OR “triplex formation” OR “triplex helix formation” OR “triplex helix structure” OR “triplex helix” OR “triplex” OR “Triplex Target Site” OR “triplex target site” OR “Triplex Target Site potential” OR “Triplex-sequence” OR “triplex technology” OR “triplex target DNA” OR “target sequence” OR “triplex-forming potential” OR “triplex-helix DNA” OR “triplex DNA” AND (“algorithm” OR “Artificial Intelligence” OR “atomic-scale modeling” OR “atomic interaction” OR “computer program” OR “computational tool” OR “computational design” OR “computer model” OR “computational modeling” OR “computer simulation” OR “computer-aided drug design” OR “chemical interactions” OR “convolutional layer-specific” OR “deep learning” OR “drug design” OR “energy models” OR “energy features” OR “enthalpy” OR “energy function” OR “electromagnetic fields” OR “electrostatic interactions” OR “force fields” OR “genetic algorithm” OR “glycosidic torsion angles” OR “hydrogen-ion concentration” OR “in silico” OR “in silico models” OR “in silico modeling” OR “in silico simulation” OR “Machine Learning” OR “mapping” OR “molecular docking simulations” OR “molecular model” OR “molecular modeling” OR “molecular recognition” OR “molecular stability” OR “molecular mechanics” OR “molecular dynamic simulation” OR “modeling bioactivity” OR “magnetic fields” OR “pharmacophore” OR “pH effect” OR “scoring accuracy” OR “structure-activity relationship” OR “software” AND NOT (“G-quadruplexes” OR “quadruplex structure” OR “tetraplex DNA” OR “quadruplex DNA” OR “in vitro” OR “in vitro techniques” OR “in vitro test” OR “in vivo”)))) AND PUBYEAR > 1979 AND PUBYEAR < 2024 AND (LIMIT-TO (SUBJAREA, “BIOC”) OR LIMIT-TO (SUBJAREA, “MEDI”) OR LIMIT-TO (SUBJAREA, “PHAR”) OR LIMIT-TO (SUBJAREA, “COMP”) OR LIMIT-TO (SUBJAREA, “NEUR”)) AND (LIMIT-TO (DOCTYPE, “ar”)) AND (LIMIT-TO (LANGUAGE, “English”))

Source: Author’s elaboration from Scopus database.

### 2.3. Bibliometric Analysis

A bibliometric analysis was performed to answer the first specific research question: “What are the publication trends, intellectual networks, and thematic patterns related to TFO in antisense and antigene strategies?”

Bibliometric data were obtained from Scopus, one of the most comprehensive scientific databases [106]. The search included publications from January 1980 to December 2023 related to computer-aided TFO design, encompassing terms associated with computational modeling, in silico methods, and rational design of ONs using the query outlines in Box 1.

**Figure 5:**
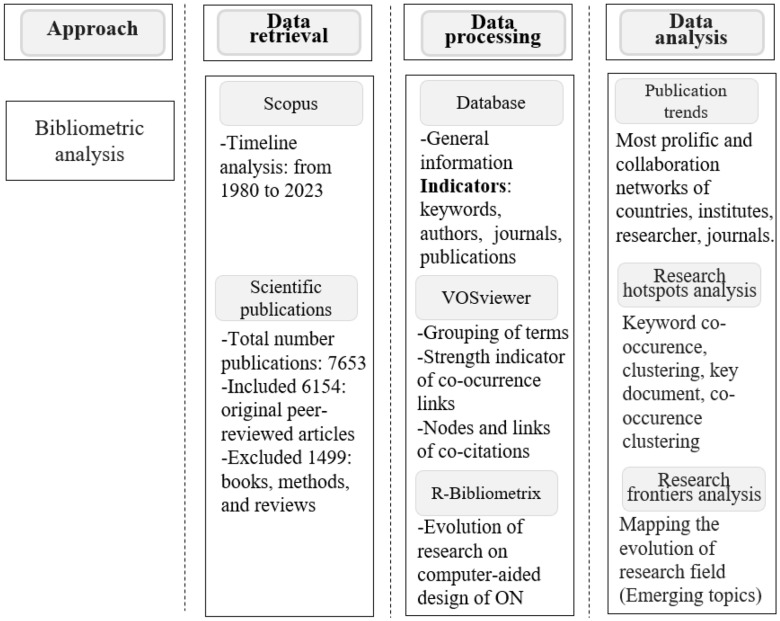
Flowchart of the bibliometric analysis applied to research on computer-aided triplex-forming oligonucleotide (TFO) design.

The search retrieved 7653 documents, all of which were downloaded with complete metadata. Figure 5 shows that only original peer-reviewed articles were included (6154); reviews, book chapters, and methods were excluded (1499) to focus on primary research contributions related to computer-aided TFO design.

The data were analyzed using VOSviewer (v1.6.19) [111] and the Bibliometrix R package (v4.2.1) [112]. The analysis focused on annual output, the most prolific authors and countries, keyword co-occurrence and thematic evolution, citation impact, and the relative prominence of antisense versus antigene strategies.

For the bibliometric component, Scopus (Elsevier) was selected as the primary data source due to its comprehensive coverage of peer-reviewed journals, detailed metadata for citation analysis, and compatibility with open-source tools such as VOSviewer and Bibliometrix. This ensured reproducibility and consistent metadata extraction across records.

In contrast, the PRISMA-guided systematic review integrated both Scopus and Web of Science to capture experimental evidence from the recent literature (2015–2024). The difference in temporal coverage between the bibliometric mapping (1980–2023) and the systematic review reflects the study’s dual design: historical mapping of research evolution versus focused synthesis of current experimental work. This methodological distinction allows complementary insight into the long-term development and present applicability of antisense and antigene strategies.

### 2.4. Systematic Review (PRISMA Framework)

This systematic review followed the PRISMA 2020 guidelines [106] to address the research question: “What are the experimental characteristics and design criteria of TFO used in antigene strategies, and how do they compare with those employed in antisense strategies, particularly in studies involving computer-aided or rational design frameworks?”

#### 2.4.1. Eligibility Criteria

We included only peer-reviewed original research articles that used computationally assisted (in silico) methods for designing, modeling, or predicting ONs targeting human genes, such as oncogenes, transcriptional regulators, or other regions of the human genome. Eligible approaches included antigene, antisense, or other gene-targeting strategies.

CRISPR–Cas9 studies were included only when the guide RNA targeted endogenous human genes, and computational tools supported the design or analysis. Studies were excluded if the ON targets were viral or bacterial, regardless of whether human cells were used as models. CRISPR–Cas9 studies were also excluded if they focused only on diagnostic applications, gene labeling, or targeting non-human sequences, or if no in silico component was involved.

Additionally, reviews, book chapters, and methods were excluded.

#### 2.4.2. Information Sources and Search Strategy

A comprehensive search was conducted in Scopus (Elsevier) [107] and the Web of Science Core Collection (Clarivate Analytics) [108], covering the period from 2014 to 2024 and downloaded in June 2025. The strategy combined controlled vocabulary terms and free-text keywords related to TFOs, ASOs, gene targeting, computational design, and in silico modeling, using Boolean operators (AND, OR, NOT), truncation, and proximity operators adapted to each database’s syntax. Filters restricted results to peer-reviewed original articles, whereas reviews, book chapters, and methodological notes were excluded; only English full-text, open-access (OA) articles were eligible. Additionally, a manual screening of the reference lists from relevant studies was conducted to identify additional eligible records. All retrieved records were imported into a reference manager, and duplicates were removed prior to screening. Database-specific queries were tailored to each platform to capture the literature on both antisense and antigene strategies, with the complete search strings for Scopus [107] and subsequently in the Web of Science Core Collection (Clarivate Analytics) [108].

- SCOPUS (Advanced Search)

(TITLE-ABS-KEY(“triplex-forming oligonucleotide” OR “triplex forming oligonucleotide” OR “Triplex-Forming Oligonucleotide” OR “Triplex Forming Oligonucleotide” OR “TFO” OR “antisense oligonucleotide” OR “ASO” OR “antisense strategy” OR “antigene strategy” OR “antigene oligonucleotide”))
AND
(TITLE-ABS-KEY(“in silico” OR “in silico model” OR “molecular modeling” OR “molecular dynamics” OR “computer-aided design” OR “computational prediction” OR “rational design” OR “structure-based design”))
AND NOT
(TITLE-ABS-KEY(“G-quadruplex” OR “quadruplex DNA” OR “quadruplex structure”))

- Web of Science Core Collection (Advanced Search)

TS = (“triplex-forming oligonucleotide” OR “triplex forming oligonucleotide” OR “TFO”
OR “antisense oligonucleotide” OR “ASO” OR “antisense strategy”
OR “antigene strategy” OR “antigene oligonucleotide”)
AND
TS = (“in silico” OR “molecular modeling” OR “molecular dynamics”
OR “computer-aided design” OR “rational design” OR “structure-based design”)
NOT
TS = (“G-quadruplex” OR “quadruplex DNA” OR “quadruplex structure”)
AND
LA = (English)

#### 2.4.3. Data Collection Process

Data from each included article were extracted by a single reviewer using a predefined template to ensure consistency across records. The process involved collecting bibliographic metadata (authors, year, journal, country), study characteristics (target gene, oligonucleotide type, mechanism of action), and methodological details relevant to computer-aided design approaches. No independent duplicate data collection was conducted, and no additional information was requested from study investigators. The extracted datasets were then cross-checked for completeness and prepared for subsequent integration with bibliometric analyses.

#### 2.4.4. Data Items

Data extracted included bibliographic metadata, target genes, oligonucleotide features, and mechanism (antisense vs. antigene). We also recorded CADD methods applied, experimental context (in vitro/in vivo), and key methodological details relevant to TFO design. Only data directly linked to computer-aided TFO design were collected; no assumptions were made about missing information.

- Effect Measures. No quantitative meta-analysis was performed due to variability in study designs and outcomes. Instead, results were described narratively, with bibliometric data presented as frequencies, percentages, and network-based measures (e.g., co-occurrence and cluster analyses). Systematic review findings were summarized using counts, proportions, and comparative tables that contrasted antisense and antigene strategies. This descriptive and comparative approach ensured consistency across datasets, providing the rationale for avoiding formal quantitative synthesis and focusing instead on mapping gaps and trends in computer-aided TFO design.
- Study Risk of Bias Assessment. No formal risk of bias tool (e.g., RoB 2.0 or ROBINS-I) [113] was used because the main goal of this study was a descriptive synthesis rather than a critical review of intervention effects. Instead, potential biases were minimized through predefined eligibility criteria (peer-reviewed original articles, English language, open-access, full-text availability), systematic screening of titles and abstracts, and duplicate removal with AteneaSIRES v1.0.3, https://ateneasires.com [55]. All included a single reviewer, who reviewed the studies to ensure consistency. Reporting bias (e.g., missing results or selective reporting) was not formally assessed, as no quantitative synthesis or outcome pooling was conducted.
- Certainty Assessment. No formal framework, such as GRADE [114], was applied to assess certainty in the body of evidence, given the descriptive and non-interventional nature of this dual bibliometric and systematic review. Instead, confidence in the findings was supported through predefined eligibility criteria, systematic screening, and metadata quality control using Bibliometrix, which collectively ensured consistency and transparency of the evidence base.
- Registration and Protocol. This systematic review was not prospectively registered in any public database (e.g., PROSPERO [115], and no pre-specified protocol was archived. Consequently, no protocol amendments were applicable. The review process was instead guided by the PRISMA 2020 statement [106], ensuring structured reporting and methodological transparency.

#### 2.4.5. Data Cleaning and Software Tools (AteneaSIRES)

All retrieved records were exported, merged, and processed to identify and remove duplicates using the open-source software AteneaSIRES v1.0.3 (https://ateneasires.com/) [55]. This platform was selected for its proven ability to detect duplicate entries through metadata harmonization, thereby reducing redundancy, minimizing the risk of bias or overrepresentation of specific studies, and ensuring transparency and reproducibility.

This method provided a strong and transparent basis for selecting evidence, ensuring that the final set of peer-reviewed articles met both quality standards and thematic relevance criteria. The number of records retrieved from each database, along with the number of duplicates identified and removed, is summarized in Table 3 (Records Retrieved and Duplicates Removed by Source).

#### 2.4.6. Data Merging and Quality Control Procedures

After removing duplicates with AteneaSIRES v1.0.3, https://ateneasires.com [55], records from Scopus [107] and Web of Science [108] were merged into a single dataset. This integration unified key metadata fields for bibliometric analysis, such as author details, publication year, source title, abstract, keywords, and citation metrics.

To ensure completeness and reliability, a quality audit was performed using the biblioAnalysis() function from the Bibliometrix R-package [112]. The audit assessed the presence or absence of key bibliographic fields and categorized data completeness as Excellent, Good, or Poor based on predefined thresholds. This step was essential for identifying structural inconsistencies and ensuring the dataset’s analytical validity before conducting descriptive or network-level bibliometric analyses.

Table 4 (Metadata Completeness of Records Retrieved from Scopus and Web of Science Assessed Using Bibliometrix in R) shows the percentage of non-missing values for each field from both sources and the final merged file. The results indicate 100% completeness for key fields, such as title, authors, year, and source, with minor gaps in keywords and affiliations, mainly in older records or due to indexing inconsistencies.

**Figure 6:**
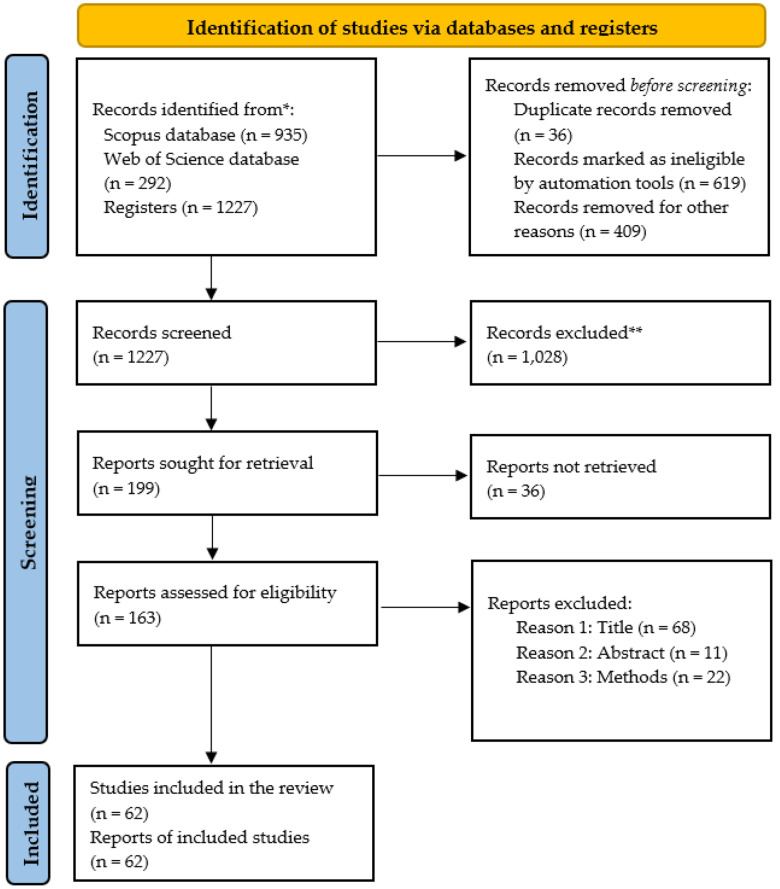
PRISMA flow diagram of study selection. This diagram summarizes the study identification, screening, eligibility assessment, and inclusion process following the PRISMA 2020 guidelines [106]. Records were retrieved from Scopus [107] and the Web of Science Core Collection [108], and duplicates were removed using the open-source software AteneaSIRES v1.0.3, https://ateneasires.com [55]. All reports sought for retrieval were successfully obtained in full text; therefore, no reports were classified as not retrieved. Reasons for exclusion at the title, abstract, and methods stages are detailed in the diagram. Each included article corresponded to a unique study; therefore, the number of studies and reports is identical. Risk of bias was minimized through predefined criteria and systematic screening. No PROSPERO registration or prior protocol available. * Records identified from bibliographic databases. ** Records excluded during screening.

After applying the eligibility criteria, data cleaning, and quality control procedures, all records moved on to the screening stage. The study selection process for this systematic review was conducted in accordance with the PRISMA 2020 guidelines [106]. Figure 6 shows a detailed visual flowchart of the identification, screening, eligibility assessment, and inclusion steps, indicating the number of records at each stage and the reasons for exclusion when relevant. In this review, each article included was related to a unique study. No multiple reports from the same investigation were found; therefore, the number of studies in the review matches the number of reports included.

The PRISMA 2020 checklist used to guide the reporting of this systematic review is provided as Supplementary Materials, reinforcing methodological transparency and alignment with international reporting standards (available at Supplementary Materials).

#### 2.4.7. Software and Analytical Tools

Bibliometric and network analyses were conducted using R 4.3.1 (R Foundation for Statistical Computing, Vienna, Austria) and Bibliometrix 4.2.0 (University of Naples Federico II, Naples, Italy). Mapping and visualization were performed with VOSviewer 1.6.20 (Leiden University, Leiden, The Netherlands). AteneaSIRES v1.0.3 (in-house development, Colombia; Registro 13-95-463, 2023) was used for bibliometric data cleaning and integration.

## 3. Results and Discussion

This section presents the results and discussion in an integrated manner, organized into two main analytical parts that reflect the dual methodology of our study. First, the bibliometric analysis [111] is reported, along with its interpretation, which highlights the mapping of research trends and the intellectual structure of the field. Second, the findings from the PRISMA 2020-guided systematic review [106] are presented and discussed, focusing on experimental evidence and comparing computational design methods in antisense and antigene strategies. Finally, an integrated subsection compares insights from both approaches, outlining implications for design practice and future directions. No meta-analysis was performed; instead, a narrative, comparative, and descriptive synthesis (antisense vs. antigen) was conducted.

### 3.1. Bibliometric Findings and Discussion

A total of 6154 original peer-reviewed articles on computer-aided ON design research were retrieved from the Scopus database, covering the period from 1980 to 2023. After removing duplicates and irrelevant document types, 5732 articles were screened by title and abstract. Notably, more than 80% of the articles originated from the United States, the United Kingdom, Japan, Germany, and China, indicating a global interest in ON research (Table 5).

The dominance of publications from the United States, the United Kingdom, Japan, Germany, and China (Table 5) focused on global disparities in research infrastructure and funding, rather than indicating exclusive scientific leadership. This geographic concentration should be viewed as a reflection of visibility and database coverage, rather than a sign of the absence of contributions from other regions. Although Scopus was selected as the primary source due to its superior metadata for bibliometric mapping, using both Scopus and Web of Science in the systematic review provided complementary evidence coverage. This clarification has been included in the Methods section to ensure that both datasets are aligned and to acknowledge that indexing in English-only sources may underrepresent non-English antigene studies.

The bibliometric analysis highlights a clear evolutionary path in research on computer-aided ON design, characterized by two distinct growth phases. The first phase (1980–2000) is foundational, characterized by moderate publication output and early research into structure–function relationships in ONs, particularly within the context of antisense frameworks. The second phase, which began in the early 2000, exhibits a significant and steady increase in publications, coinciding with advancements in molecular biology, high-throughput sequencing, and computational chemistry. This expansion indicates the maturing of computational methods and their integration into experimental processes for ON development.

The intellectual structure of the field, as revealed through keyword co-occurrence networks and thematic mapping, showed a clear dominance of antisense technologies. Terms such as “siRNA,” “ASOs,” and “gene silencing” appear in over 60% of the top 100 articles, positioning them at the core of thematic clusters and underscoring their central role in shaping the knowledge base of the discipline (Table 6).

**Figure 7:**
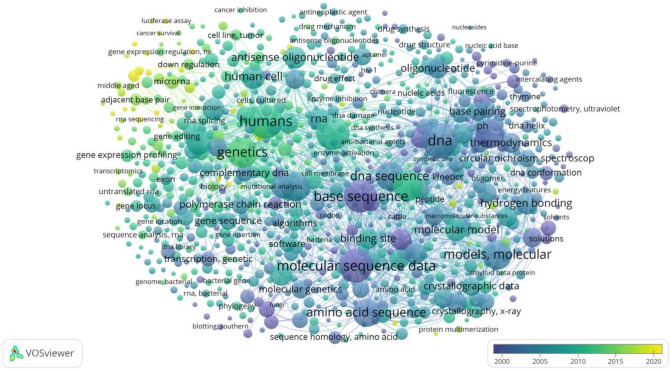
VOSviewer-based temporal visualization of keywords in computer-aided triplex-forming oligonucleotides (TFOs) design research (1980–2023). This visualization, created with VOSviewer, is based on mathematical expressions that determine node color, size, and proximity. Larger nodes represent keywords linked to a greater number of publications. Nodes placed closer together show stronger co-occurrence relationships. Central nodes represent mature and influential topics, typically colored green (core/essential) or purple (highly impactful). Peripheral nodes, often in yellow, indicate emerging or trending issues. Source: Author’s elaboration using VOSviewer software, based on the Scopus dataset.

In contrast, “antigene” and “triplex DNA” are far less frequently detected in only 15% and 12% of records, respectively, and cluster on the right side of the map, typically co-occurring with less-cited or older publications (Figure 7), reflecting the marginal role of antigene strategies and highlighting the technological gap for advancing rational design methods.

Keyword analysis of 1078 terms and over 130,000 co-occurrence links further supported this imbalance. The most common unigrams included DNA, gene, sequence, structure, antisense, target, oligonucleotides, interaction, binding, and modeling, while frequent bigrams were targeting sequence, molecular dynamics, nucleic acid, binding sites, and crystal structure. Emerging trigrams featured antisense oligonucleotides (ASOs), non-coding RNAs (lncRNAs), RNA interference (RNAi), and triple helix formation (Table 6). These distributions highlight the centrality and diversification of antisense applications, whereas terms related to triplexes or antigens remain rare and confined to niche or declining quadrants.

**Figure 8:**
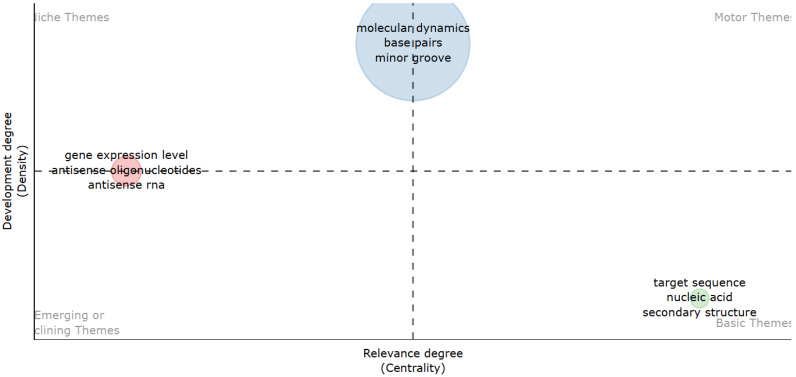
Thematic map of research on computer-aided triplex-forming oligonucleotides (TFOs) design for period 1 (1980–2013). The thematic map classifies research topics into four quadrants based on their centrality and density: Quadrant 1 (upper right): Motor themes—well-developed and central topics, such as molecular dynamics (MD), base pairs, and mirror groove (represented by the blue bubble labeled “molecular dynamics (MD)”). Quadrant 2 (lower right): Basic themes—important but less internally developed topics, including target sequence, nucleic acid, and secondary structure (green bubble labeled “target sequence”). Quadrant 3 (upper left): Niche themes—specialized topics that are well-developed but peripheral. Quadrant 4 (lower left): Emerging or declining themes—less developed and marginal topics. In this thematic map, terms such as gene expression level, antisense oligonucleotides (ASOs), and antisense RNA (red bubble labeled “gene expression level”) appear to span both Quadrant 3 and Quadrant 4. Thematic maps of research on computer-aided TFO design for periods 1980–2013 and 2014–2023. Bubble size = keyword frequency; color = cluster identity. Quadrants represent motor (upper right), basic (lower right), niche (upper left), and emerging/declining (lower left) themes. Source: Author’s elaboration using R-Bibliometrix (Scopus dataset). Source: Author’s elaboration using the R-Bibliometrix package, based on the Scopus dataset.

**Figure 9:**
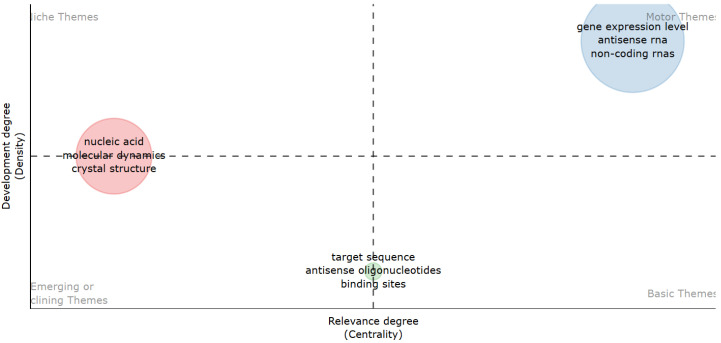
Thematic map of research on computer-aided triplex-forming oligonucleotides (TFOs) design for period 2 (2014–2023). The thematic map categorizes research topics into four quadrants based on their centrality and density. Quadrant 1 (upper right): Motor themes—comprises well-developed and central topics, such as gene expression levels, antisense RNA, and non-coding RNAs (represented by the blue bubble labeled “gene expression level”). Quadrant 2 (lower right): Basic themes—important but less internally developed topics, including target sequence, antisense oligonucleotides (ASOs), and binding sites (green bubble labeled “target sequence”). Quadrant 3 (upper left): Niche themes—specialized topics that are well-developed but peripheral. Quadrant 4 (lower left): Emerging or declining themes—less developed and marginal topics. In this thematic map, terms such as nucleic acid, molecular dynamics (MD), and crystal structure (red bubble labeled “nucleic acid”) appear to span both Quadrant 3 and Quadrant 4. Bubble size = keyword frequency; color = cluster identity. Quadrants represent motor (upper right), basic (lower right), niche (upper left), and emerging/declining (lower left) themes. Source: Author’s elaboration using the R-Bibliometrix package, based on the Scopus dataset.

**Figure 10:**
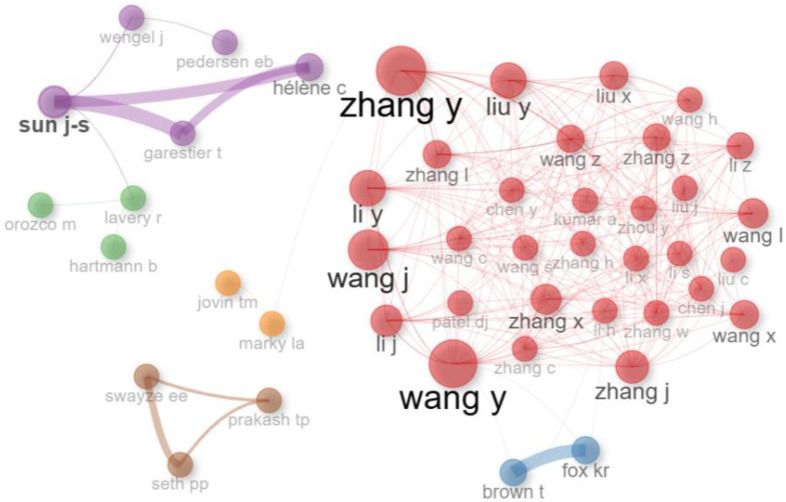
Collaborative networks of the author in the computer-aided design of triplex forming oligonucleotides (TFO). Cluster visualization of co-authorship analysis created using VOSviewer software [111]. Six clusters are distinguished by different colors (blue, brown, green, purple, red, and yellow). Each cluster contains various nodes, and the size of each node indicates the number of citations. Co-authorship network in computer-aided TFO design. Colors denote collaborative clusters identified by VOSviewer; node size = number of citations per author. Larger nodes indicate higher influence; thicker links represent stronger collaboration intensity. Source: Author’s elaboration using the R-Bibliometrix package [112], based on data extracted from the Scopus dataset.

To illustrate the evolution of research themes in computer-aided TFO design, the analysis was split into two time periods using thematic mapping. In the thematic maps (Figure 8 and Figure 9), the size of the bubbles indicates the frequency of keyword appearance, while the color represents each topic cluster created by the R-Bibliometrix algorithm. Centrality indicates the degree to which a theme is connected to others, and density reflects its internal cohesion. Quadrants I–IV represent, respectively, motor, basic, niche, and emerging or declining themes. In the co-authorship network (Figure 10), each color represents a collaboration cluster, and node size shows the number of citations per author, highlighting both productivity and influence in the field.

Period 1 (1980–2013) revealed that “molecular dynamics (MD),” “base pairs,” and “mirror groove” were the most established and advanced research themes. These topics occupied Quadrant 1 (motor themes), showing they were central and strongly connected within the research network. In contrast, “gene expression level,” “antisense ON,” and “antisense RNA” appeared as peripheral or emerging themes (Quadrants 3 and 4), indicating limited development and visibility at that time (Figure 8).

Period 2 (2014–2023) marked a shift. The same antisense-related themes transitioned to Quadrant 1, becoming core topics in the field. This evolution demonstrates how antisense applications have become a focal point in ON design research. Meanwhile, structural and antigene-related terms, such as “nucleic acid,” “MD,” and “crystal structure,” became less central or declined as themes (Figure 9).

The temporal analysis reinforces this asymmetry: antisense-related topics evolved from peripheral (Figure 7) or emerging issues in the earlier period (1980–2013; Figure 8) to core motor themes in the most recent decade (2014–2023; Figure 9). Conversely, antigene-specific concepts such as nucleic acid, molecular dynamics (MD) linked to triplex stability, and crystal structure remained marginal, failing to transition into central clusters. Together, these findings show that, despite their mechanistic promise, antigene approaches have not achieved sustained integration into mainstream research agendas, in stark contrast to the dynamism and consolidation of antisense strategies. This highlights that antisense applications currently drive research, while triplex-specific or antigene strategies remain underused and mostly unexplored.

The dataset from Scopus included 24,630 authors, with highly cited individuals often having strong collaborative networks that improve publication rates. We use the Leiden algorithm [116] and the R-Bibliometrix package [112] to analyze this network. Applying the Leiden algorithm, we identified seven clusters of co-authors, with the red cluster being the most productive and internationally interconnected (Figure 10). Key contributors were mainly affiliated with institutions in Singapore, China, the UK, and the US. Notable authors included Zhang Y. (51 publications) [117], Wang Y. (47 publications) [118], and Wang J. (42 publications) [119], all of whom were within the red cluster (Table 5). Wang, Y., had the highest citation count at 530 (red cluster) [120], followed by Seth, PP, with 355 (pink cluster) [121], and Sun, J-S, with 314 (purple cluster) [71] (Figure 10; Table 5). In 2016, 25 researchers from Isis Pharmaceuticals, including Prakash TP, Swayze, EE, Seth, PP (pink cluster), led a study on N-acetyl-galactosamine-conjugated antisense ONs, which was cited 38 times [122]. Wang H-Z and 20 other authors (red cluster) developed a gene resequencing chip in 2010, cited at least 10 times [123]. The same year, Li Y and 20 other authors (red cluster) led research on a syndrome caused by loss-of-function mutations in chondroitin synthase 1, which received 75 citations [124]. Zhang K and 18 authors (red cluster) studied the SARS-CoV-2 RNA genome in 2021, earning 46 citations [125]. Lavery R (2010) and 17 authors (green cluster) conducted a systematic MD study on B-DNA conformations, a landmark work cited 258 times [126]. In 2020, Zhang J and 17 collaborators (red cluster) investigated the long non-coding RNA HOTAIRM1, cited 10 times [127]. Hsu PH and colleagues (2013) defined DNA-targeting specificity of RNA-guided Cas9 nucleases, a seminal genome-editing study cited over 3174 times [128]. Lui Y and colleagues (2013) applied Support Vector Machine to classify coding and non-coding transcripts, a pioneering machine learning approach in genomics, which has been cited over 1180 times [129] (Figure 10).

The most cited works in the corpus include VSEARCH, an open-source tool for processing nucleotide sequence data in metagenomics and genomics [130], cited 4787 times; Cas9 guide RNA architecture by Hsu et al. [128], cited 3174 times, which contributed to the development of CRISPR-based genome editing tools and introduced a web platform for sgRNA design and off-target analysis; and DrugBank [131], cited 2625 times, a comprehensive database that integrates chemical and protein information to support in silico drug discovery, docking, and pharmacological prediction.

**Figure 11:**
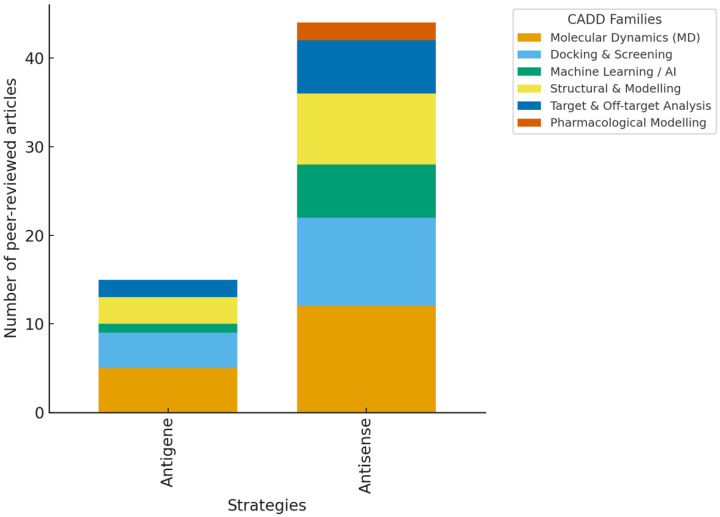
Distribution of computer-aided drug design (CADD) methods by mechanism (Antisense vs. Antigene) grouped into six families. Antisense strategies showed diverse applications, primarily through docking and molecular dynamics (MD), whereas antigene approaches mainly relied on triplex-specific modeling. Study outcomes were summarized descriptively by the oligonucleotide (ON) type and CADD method; no pooled effect estimates were calculated due to study heterogeneity. Confidence in the evidence remains uneven: robust for antisense strategies but limited and variable for antigene studies.

### 3.2. Systematic Review Findings and Discussion

A total of 935 full-text, original peer-reviewed articles were retrieved from the Scopus [107] and Web of Science [108] datasets for eligibility assessment. After screening the titles, abstracts, and methods, 62 studies met the inclusion criteria and were included in the final review (see Figure 6: PRISMA Flow Diagram).

For each included study, outcomes were summarized descriptively in structured tables. These tables documented study characteristics, targeted genes, ON types, mechanisms of action, and computational methods used. No pooled effect estimate or confidence intervals were calculated due to heterogeneity across study designs; instead, individual findings were narratively compared to highlight methodological trends and differences between antisense and antigene strategies, providing a structured basis for the synthesis presented below.

Of the full-text reports assessed after duplicate removal with AteneaSIRES v1.0.3, https://ateneasires.com [55], several were excluded despite initially appearing to meet the eligibility criteria. The main reasons for exclusion were lack of in silico methods (n = 11), studies targeting non-human or viral/bacterial sequences (n = 9), diagnostic- or labeling-focused CRISPR–Cas9 applications without therapeutic orientation (n = 5), and records that were reviews, methods-only, or editorials (n = 7). These exclusions are consistent with the predefined criteria outlined in Section 2.3 of the Methods. The full process is summarized in the PRISMA 2020 flow diagram (Figure 6).

No formal, study-level risk of bias assessments (e.g., RoB 2.0) [113] were performed, given the descriptive scope of this review. Instead, potential biases were mitigated through predefined eligibility criteria and systematic screening; further limitations regarding risk of bias are discussed below in this section.

**Figure 12:**
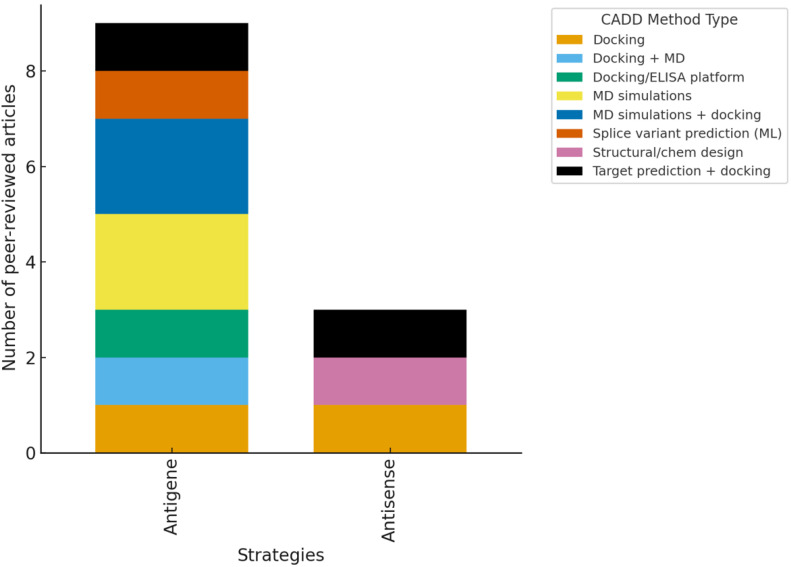
Distribution of computer-aided drug design (CADD) methods used in triplex-forming oligonucleotide (TFO) studies targeting cancer-related genes, broken down by strategy (Antisense vs. Antigene). Antigene strategies were more common, mainly relying on docking and triplex-stability modeling, especially in *c-MYC* and *KRAS* promoters. Conversely, antisense studies used fewer but more standardized computational methods. Study outcomes were summarized descriptively by the gene, oligonucleotide (ON) type, and CADD method; no pooled effect estimates were calculated due to study heterogeneity. Confidence in the evidence remains uneven: robust for antisense strategies but limited and variable for antigene studies.

After systematically identifying, screening, and assessing the eligibility of the studies, the final set of original peer-reviewed articles was analyzed to describe the use of CADD methods in oncology ON-based research. The analysis was categorized by strategies—Antisense and Antigene—and further examined for studies targeting TFOs in cancer-related genes. The results, which highlight the dominant role of CADD methods in ON research, are summarized below, supported by distribution figures and references to the individual studies included in the dataset. The synthesis of results was performed descriptively, aggregating study-level data into comparative distributions of antisense versus antigene strategies, which are visualized in Figure 11 and Figure 12.

The synthesis included heterogeneous studies, combining in-silico modeling with limited in vitro evidence. Potential sources of bias were addressed with eligibility criteria and systematic screening, although no formal risk assessment tool was employed. No quantitative meta-analysis was undertaken; instead, results were summarized descriptively and displayed in Figure 11 and Figure 12. Variability was primarily related to gene targets (e.g., c-*MYC*, *KRAS*), ON chemistry, and computational methods. Sensitivity analyses were not conducted, but the descriptive synthesis provides a coherent overview of antisense and antigene strategies. No formal assessment [113] of reporting bias was performed because the studies included were highly heterogeneous, and no registered protocols were available for comparison. Nonetheless, all eligible full-text articles were retrieved and analyzed, and no evidence of selective outcome reporting was found during data extraction.

Given the descriptive nature of this review, no formal GRADE assessment [114] was conducted. Confidence in the evidence is limited due to the small number of antigene-focused studies, variability in methodological quality, and the lack of standardized reporting. Conversely, evidence supporting antisense strategies is more consistent, reflecting broader experimental validation and clinical development.

This section presents the results in context, notes limitations, and summarizes implications.

The distribution analysis revealed that antisense strategies mainly drive CADD in ON research, with antigene approaches (Figure 11). Within the antisense category, the most common computational methods used were molecular docking, MDS, and QSAR modeling. Notably, molecular docking was employed in studies by Ejlersen et al. (2017) [132] and Kasuya et al. (2018) [133], which helped to predict ON–target RNA interactions and improve binding affinity. Similarly, MDS were widely used to evaluate the structural stability of antisense RNA duplexes under physiological conditions, as reported by Zaniani et al. (2021) [134] and others. The results are summarized below, supported by the distribution figures (triangular distribution figures that depict the frequency of method usage) and accompanied by references to the individual studies included in the dataset.

Across all included studies, recurrent target genes were identified, reflecting a focus on modulating oncogenes and regulating pathways specifically. In antisense strategies, the most frequently targeted gene was *MALAT1* (12.5%) [135], KRAS (10.4%) [136], *BCL2* (8.3%), *MYC* (8.3%), *VEGF* (9.1%) [137], and *HOTAIR* (6.2%) [127]. These genes are crucial in cancer research because they influence cell growth, survival, and spread. They are often selected after computational screening to enhance binding site accessibility, increase hybridization efficiency, and minimize off-target effects [138]. In antigene strategies, dominant targets included *c-MYC* (18.2%) [23], *KRAS* (13.6%), *BCL2* (6.8%), and *HER2/neu* (6.8%) [29], often located in promoter regions capable of forming triplex structures. Such regions were prioritized using in silico TTS prediction and molecular docking workflows [137,139]. This focus on a small set of well-understood genes spotlights both the advancement of CADD-supported ON design pipelines and the clinical significance of these targets in cancer and other diseases.

For antigene strategies, molecular modeling—including triplex stability prediction and Hoogsteen base-pair analysis—has become the leading CADD method. Studies by Pabón-Martínez et al. (2017) [23] and Ni et al. (2023) [139] used in silico TTS mapping to identify genomic regions suitable for TFO binding. In these cases, the computational method directly guided the selection of ON sequences, improving target specificity and minimizing off-target effects.

When limiting the dataset to TFO studies involving cancer-related genes (Figure 12), antigene strategies predominated, with molecular docking and triplex stability prediction as the main tools. This pattern reflects the focus on modulating oncogene expression by targeting sequence-specific TFOs to promoters or coding regions capable of forming triple helices. Representative works include those targeting c-*MYC* and *KRAS* promoters [136], where computational analyses were essential for assessing the triplex formation potential and enhancing the ON thermodynamic profiles [140].

Overall, these findings emphasize the practical implications of our research. While antisense approaches benefit from a wide range of CADD methods that optimize RNA binding, antigene strategies depend heavily on triplex-specific modeling to guide their design. Both approaches demonstrate the crucial role of computational pipelines in accelerating the discovery of ON drugs and enhancing experimental validation, making our work highly relevant and engaging for the field. A larger proportion of antisense studies (78%) compared to antigene (22%) not only reflects current research trends but also relates to practical differences in computational versus experimental reproducibility. In antisense designs, CADD-based predictions, particularly docking and MD-derived binding affinities, demonstrate strong consistency with in vitro hybridization and inhibition results, facilitating the optimization and clinical translation of these designs. Conversely, antigene TFOs still exhibit lower predictability because their triplex stability depends on factors such as pH, ionic strength, and chromatin context, which most existing CADD platforms only partially simulate. This technical limitation contributes to the lower success rate and slower progress of antigene strategies, highlighting the need for integrated CADD pipelines that include triplex-specific energetic and structural constraints, which are still imperfectly modeled computationally—thereby limiting predictive success and clinical application.

Our PRISMA review of the 62 original peer-reviewed articles included underscores that antisense strategies using a mature set of computational tools are more diverse and advanced in antisense workflows, employing molecular docking, MD, and QSAR/QSPR [102] methods, supported by standard chemistry and software (Figure 11). Antigene designs depend on triplex-stability modeling, often without fully integrating it into accessible CADD platforms; their research mainly relies on specialized triplex-stability modeling [141] and Hoogsteen pair assessments that focus on biophysical feasibility (such as triplex formation rules, pH-dependent C^+^·G–C triplets, and constraints of polypurine targets) [142] before proceeding on to downstream optimization. Notably, among the subset targeting cancer-related genes, antigene strategies are more common, emphasizing the mechanistic compatibility of TFOs in promoter-region engagement (e.g., *c-MYC* [23], *KRAS*, *VEGF*, *BCL2*, *ETS2* [139]) (Figure 12), but also indicating the need for more standardized tools that accurately reflect physiological triplex behavior. The decline in recent antigene studies, along with unresolved methodological gaps, underscores the gap compared to antisense research and paves the way for future discussions on design practices and directions.

### 3.3. Integrative Insight Across Methods

Our comprehensive bibliometric and PRISMA analyses reveal a significant imbalance between ASOs and TFOs as therapeutic options. ASOs have achieved greater regulatory and translational maturity, 18 FDA/EMA/MHLW-approved drugs (e.g., Spinraza^®^ for spinal muscular atrophy) [46], and other orphan diseases (Table 1). This dominance comes from strong industrial pipelines and proven therapeutic chemistry, reinforcing antisense’s core intellectual position. Among these 18 approved therapeutics, ASOs primarily work through mechanisms such as mRNA degradation via RNase H [21] or RISC [37], splice modulation [42], and translation block [48], which are commonly used to treat genetic diseases. Translatability barriers are rare, having been reported in only two aptamer-based cases with highly specific functions (Table 1). Alongside a growing clinical pipeline of gapmer or LNA ASO candidates in Phase I–III trials [143], it is driving both academic interest and commercial investment.

TFOs used as an antigene strategy face substantial methodological challenges [50], which explains their limited presence in bibliometric studies. This limitation is rooted in the chemical and structural nature of Hoogsteen hydrogen bonding, which governs triplex formation and requires homopurine–homopyrimidine tracts for stable recognition. Current CADD platforms, optimized for canonical Watson–Crick duplexes, lack accurate parameterization for protonated cytosines, local torsional dynamics, and noncanonical base stacking that define triplex geometry. Consequently, existing molecular docking and molecular dynamics engines cannot yet fully simulate the pH-dependent and sequence-constrained environment of triple helix formation.

Nevertheless, there is renewed momentum supporting an antigene strategy. Emerging methodologies, including constant-pH MD for modeling triplex stability under physiological conditions [144], chromatin-aware target site prediction, and AI-driven multi-objective optimization [145], are designed to overcome historical limitations caused by structural requirements such as homopurine–homopyrimidine tract necessity [51], poor thermal and biological stability [52], susceptibility to pH and ionic fluctuations [53], and limited cellular accessibility [146]. These physicochemical and delivery limitations have historically prevented antigene TFOs from gaining clinical approval, despite a strong mechanistic rationale. The integration of next-generation CADD, chemical stabilization (e.g., base and backbone analogs), and improved delivery vectors now offers a viable path toward translational validation. These advances enable new applications in gene therapy [76], synthetic biology [75], and epigenetic regulation [141], positioning TFOs as promising therapeutic agents [9] alongside ASOs.

The PRISMA evidence supports a method-rich antisense landscape and a focused but under-explored, antigene niche. Bridging this gap by adapting antisense CADD best practice, particularly those based on docking, molecular dynamics, and AI-driven optimization (see Figure 11) [65], to the physicochemical constraints of triplex formation offers a clear and technically grounded path toward revitalizing the antigene approach and integrating it into the next generation of nucleic acid therapeutics.

Unlike earlier reviews that broadly examined oligonucleotide therapeutics and their pharmacological development, such as Vasquez and Glazer (2002) [8], Khvorova and Watts (2017) [28], and Hasselgren and Oprea (2024) [1], the current study uniquely combines bibliometric mapping with a PRISMA-based systematic review to identify the specific methodological and translational gaps in antigene TFO design. Whereas previous works emphasized antisense or siRNA clinical trajectories, our analysis highlights the persistent structural and computational barriers that have limited the advancement of antigene TFOs, while proposing CADD- and AI-based frameworks to bridge this gap.

### 3.4. Implications for Design Practice and Future Research

Based on previous insights, we suggest a streamlined, narrative roadmap: designers could identify cancer-relevant gene regions capable of forming triplexes, such as promoter polypurine tracts for antigene approaches and accessible transcripts for antisense strategies. Using TTS prediction tools and chromatin data improves biological relevance. Designer sequences must ensure specificity, nuclease resistance, and compatibility with physiological conditions [53], verified through docking [147], MDS under physiological conditions [23], and binding free-energy calculations [32].

Next, machine learning models should be trained using features derived from sequence motifs [148], docking/MD descriptors [147], and nuclease-based gene-editing tools, which are being explored for therapeutic applications. In this context, CADD optimization of TFOs can complement and enhance CRISPR–Cas9 technology. Both systems rely on programmable base recognition and face similar challenges, such as chromatin accessibility, sequence-dependent energetics, and off-target effects. Integrating triplex modeling principles into CRISPR targeting algorithms could improve guide RNA accuracy, while CRISPR-based genome-mapping tools might enhance TFO target-site prediction in regulatory regions, thereby creating a mutual benefit between antigene and gene-editing approaches.

Notably, machine learning platforms such as eSkip-Finder [149] and ASOptimizer [150] have enhanced ASO design by predicting effectiveness and guiding chemistry choices, and recent efforts combine molecular modeling with ML for ligand development [151]. Training these models allows candidates to be quickly classified based on their affinity, specificity, and stability. Finally, well-defined acceptance criteria, such as adequate binding affinity at physiological pH [152], ionic strength, high specificity, and degradation resistance, should determine which ON antigene candidates proceed to laboratory validation.

In practical terms, our synthesis highlights a core set of computational resources and molecular targets that can inform future design of antigene TFOs. Among CADD tools, Triplexator [153], LongTarget [154], Fasim-LongTarget [146], and TriplexAligner [155] remain the most reliable for in silico prediction of triplex sites [152]. In contrast, docking engines (AutoDock, HADDOCK) [2] and molecular dynamics packages (GROMACS, AMBER) [32] are crucial for assessing the stability of TFO–DNA interactions under physiological conditions. Machine learning platforms such as ASOptimizer [150] and eSkip-Finder [149] offer complementary capabilities for model training and sequence refinement.

From a biological perspective, the most consistent and experimentally validated targets for antigene TFOs include c-*MYC* [23], *KRAS* [156], *BCL2* [82], *VEGF* [157], and *HER2/neu* [29], all of which are characterized by promoter regions rich in homopurine tracts suitable for triplex formation. Integrating TTS prediction, chromatin accessibility data, and constant-pH MD simulations provides a rational framework for prioritizing new candidate regions. Collectively, these elements constitute a reproducible pipeline that can strengthen the translational potential of antigene strategies in computational and experimental therapeutics.

In future developments, advanced machine learning architectures can further refine antigene TFO design. Convolutional neural networks (CNNs) are well-suited for detecting spatial and motif-based patterns within triplex-forming sequences [158], whereas recurrent neural networks (RNNs) [159] and long short-term memory (LSTM) models can capture positional dependencies across nucleotide contexts [160]. Recently, transformer-based architectures [161] trained on large nucleotide [158] corpora have shown better performance in predicting nucleic acid binding and folding dynamics [162]. Combining these deep-learning models with CADD descriptors—such as docking scores, thermodynamic profiles, and MD-derived features—could enable multi-objective optimization frameworks that simultaneously improve TFO stability, selectivity, and target accessibility.

### 3.5. Limitations of the Present Study

The restriction to full-text, English-language, open-access studies ensured methodological transparency and reproducibility but may have introduced selection bias, particularly by underrepresenting non-English or paywalled articles. While this limitation might influence the apparent magnitude of the antisense–antigene disparity, it does not alter the overall conclusion that antisense strategies remain methodologically and clinically more advanced. This review did not apply a formal risk of bias tool [113] or GRADE framework for certainty assessment [114]. A single reviewer performed all screening and data extraction. The study lacked a registered protocol (e.g., PROSPERO [115]), and no amendments were applicable. These factors, together with the absence of subgroup or sensitivity analyses, may limit reproducibility and critical appraisal; however, they remain consistent with the descriptive scope of this dual-method study.

### 3.6. Integrated Forward Path: Toward Therapeutic TFOs

**Figure 13:**
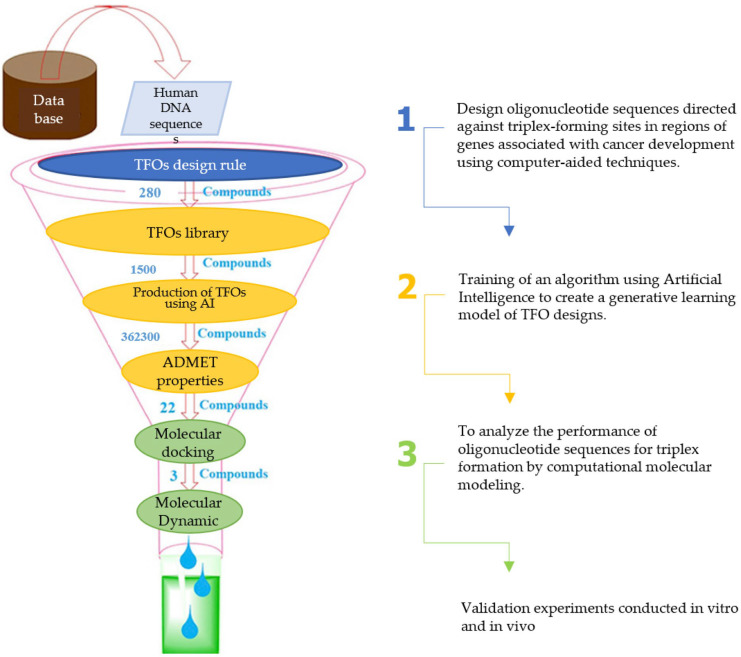
Integrative computational pipeline for antigene triplex-forming oligonucleotide (TFO) design. The diagram illustrates a step-by-step in silico workflow—from target site prediction and AI-based optimization to docking and molecular dynamics (MD) validation—that filters candidate sequences to identify the most stable and specific TFO for further experimental validation. AI (Artificial Intelligence); ADMET (absorption, distribution, metabolism, elimination/excretion, toxicity).

The research landscape of computer-aided TFO design in antisense and antigene strategies requires a systematic and unified framework. Our dual-method analysis highlights the importance of combining bibliometric insights, canonical triplex rules, and translational priorities into a cohesive design process. Traditional CADD approaches have often fallen short because the generated compounds lack essential therapeutic properties; however, AI can be used to improve these characteristics [1], and deep learning now provides the ability to accelerate design-based DNA:RNA TFOs [117], evaluate large quantities of molecules quickly, enhance accuracy, and improve translational outcomes [163]. Iterative pipelines that incorporate recent chemical advances (e.g., backbone analogs and base modifications) [164], pH-ion dynamics [165], AI-driven optimization [2,145], in silico ADMET property calculations to analyze pharmacokinetics profiles [3,12], combined with cutting-edge iterative docking–MD cycles modeling that now accurately model triplex thermodynamics [166], can substantially improve stability, specificity, and delivery. Structured as a funnel-shaped workflow automation (see Figure 13), these steps enable efficient filtering to prioritize a small set of candidates TFO sequences for synthesis and functional testing, reducing attrition in later stages. This roadmap offers both conceptual and methodological guidance, combining proven antisense design practices with actionable strategies to reinforce antigene approaches, ultimately enhancing the potential of therapeutic TFOs with solid mechanistic and translational support.

### 3.7. Final Contributions

This study provides a thorough, evidence-based review of the experimental landscape related to computer-aided TFO design in the context of antisense and antigene therapeutic strategies. By combining bibliometric mapping (see Figure 5) with a PRISMA-guided systematic review (see Table 2; Figure 6), we established a dual-method framework that captures both scientific progress and experimental evidence in the field. Our findings reveal a clear regulatory and translational imbalance: antisense strategies demonstrate clinical maturity with 18 FDA/EMA/MHLW-approved drugs (see Table 1) and a robust clinical pipeline, whereas antigene approaches remain delayed due to inconsistent adherence to canonical triplex design rules and limited validation. The bibliometric analysis of 24,630 authors further mapped key collaborative networks that shape research pathways (see Table 5; Figure 10). Finally, we underscore the potential of CADD tools (see Figure 11; Figure 12), particularly MD, docking, and AI-driven optimization, to bridge current gaps and reintroduce antigene strategies into therapeutic development (see Figure 13).

## 4. Conclusions

This study reveals an evidence-based overview of the experimental landscape related to computer-aided TFO design in antisense and antigene therapeutic strategies, combining bibliometric mapping with a PRISMA-guided systematic review. Our findings show a clear imbalance: antisense approaches lead the field’s intellectual and translational areas, supported by regulatory approval of 18 drugs and strong CADD pipelines, contrasted with antigene strategies that remain underrepresented. Antigene strategy faces challenges such as limited adherence to canonical triplex rules, poor biochemical stability, and limited in vivo validation. Although AI-driven optimization for docking and MD offer promising possibilities, the current evidence suggests that antigene TFO design remains an emerging, technically limited approach that requires significant methodological development before it can be applied in clinical settings.

## Figures and Tables

**Table 2:** Comparative overview of Scopus and Web of Science for systematic reviews on computational TFO design.

**Feature**	**Scopus (Elsevier)**	**Web of Science (Clarivate)**
Use	Bibliometric analysis and PRISMA-based systematic review	PRISMA-based systematic review
Coverage	Broad multidisciplinary (biomedicine, pharmacology, chemistry, bioinformatics)	Highly curated, multidisciplinary; emphasis on high-impact journals
Peer-review filtering	Yes—by document type and journal source	Yes—by category and inclusion in JCR
Peer-review language	English	English
Publication year	2015–2025	2015–2025
Primary journal metric	CiteScore: Average citations per document over 4 years	Impact Factor (IF): Avg. citations to articles from the previous 2 years
Normalized metric	SNIP: Field-normalized impact per paper	JCI: Journal Citation Indicator (baseline = 1.0)
Prestige-weighted metric	SJR: Source prestige + network influence	Eigenfactor Score: Network influence, excluding self-citations
Per-article influence	Not available	Article influence score: Derived from Eigenfactor
Immediacy metric	Not available	Immediacy index: Same-year citations
Quartile ranking	Q1–Q4 based on CiteScore or SJR	Q1–Q4 based on JCR categories
Years covered	From 1996 onwards	From 1900 onwards
Open access indicator	Integrated filters and metric display	Integrated in JCR with OA tags
Citation database source	Scopus Citation Index	Web of Science Core Collection
Relevance for TFO design	High—Ideal for in silico design and bioinformatics applications	High—Strong for both historical and current experimental TFO research

Abbreviations: JCR (Journal Citation Reports), IF (Journal Impact Factor), SNIP (Source Normalized Impact per Paper), JCI (Journal Citation Indicator), SJR (SCImago Journal Rank), OA (Open Access), TFO (triplex-forming oligonucleotide).

**Table 3:** Summary of records retrieved and duplicates removed by source.

Source	Originally Downloaded Data	Deleted Data	Merged Data	% Merged Data
Scopus	128	36	92	56.4
Web of Science (WoS)	71	0	71	43.6
Total	199	36	163	100.0

**Table 4:** Metadata completeness of records retrieved from Scopus and Web of Science was assessed using Bibliometrix in R.

Metadata	Description	^a^ Scopus			^b^ Web of Science (WoS)			^c^ Meged Data		
		Missing Counts	Missing %	Status	Missing Counts	Missing %	Status	Missing Counts	Missing %	Status
AB	Abstract	0	0.00	Excellent	0	0.00	Excellent	0	0.00	Excellent
C1	Affiliation	0	0.00	Excellent	0	0.00	Excellent	0	0.00	Excellent
AU	Author	0	0.00	Excellent	0	0.00	Excellent	0	0.00	Excellent
DI	DOI	0	0.00	Excellent	0	0.00	Excellent	0	0.00	Excellent
DT	Document Type	0	0.00	Excellent	0	0.00	Excellent	0	0.00	Excellent
SO	Journal	0	0.00	Excellent	0	0.00	Excellent	0	0.00	Excellent
LA	Language	0	0.00	Excellent	0	0.00	Excellent	0	0.00	Excellent
PY	Publication Year	0	0.00	Excellent	0	0.00	Excellent	0	0.00	Excellent
TI	Title	0	0.00	Excellent	0	0.00	Excellent	0	0.00	Excellent
TC	Total Citation	0	0.00	Excellent	0	0.00	Excellent	0	0.00	Excellent
ID	Keywords Plus	2	1.56	Good	4	5.63	Good	5	3.07	Good
DE	Keywords	36	28.12	Poor	31	43.66	Poor	56	33.74	Poor

Completeness of metadata on peer-reviewed articles: ^a^ 128; ^b^ 71; ^c^ original size 199 and 36 duplicates deleted.

**Table 5:** Top high-impact authors in research on computer-aided triplex-forming oligonucleotide (TFO) design, 1980–2023.

Author	Country	Institution	Number of Articles Issued	Global Citations	Average Co-Authorship	^a^ Cluster
Zhang Yu	Singapore	Nanyang Technological University	51	283	5	Red
Wang Y	England	University of Southampton	47	530	5	Red
Wang J	USA	University of California	42	146	8	Red
Liu Y	Singapore	Nanyang Technological University	36	145	4	Red
Seth, PP	USA	Isis Pharmaceuticals	34	355	10	Brown
Li Y	China	Peking University	34	186	6	Red
Zhang X	USA	University of California	31	291	3	Red
Wang L	China	Peking University	30	116	6	Red
Sun J-S	France	Muséum National d’Histoire Naturelle	27	314	2	Purple
Hélène C	France	Muséum National d’Histoire Naturelle	24	55	2	Purple
Wengel J	Denmark	University of Southern	23	43	3	Purple
Swayze, EE	USA	Isis Pharmaceuticals	15	323	7	Brown

^a^ The clusters are visualized and explained below.

**Table 6:** Top high-frequency keywords and centrality in research on the computer-aided design of triplex-forming oligonucleotides (TFOs), 1980–2023.

^a^ Unigrams	Occurrences	Pagerank_Centrality	^b^ Bigrams	Occurrences	Pagerank_Centrality	^c^ Trigrams	Occurrences	Pagerank_Centrality
DNA	2386	0.013	Target sequence	580	0.018	Molecular dynamics simulations	146	0.048
Sequence	2132	0.012	Gene expression level	488	0.020	Polymerase Chain Reaction	129	0.019
Structure	1848	0.011	Molecular dynamics	362	0.017	Nuclear Magnetic Resonance	94	0.019
Antisense	1771	0.009	Nucleic acid	307	0.011	Amino acid residues	79	0.008
Target	1770	0.010	Mirror groove	273	0.013	Nucleic acid research	52	0.008
Gene	1673	0.009	Antisense oligonucleotides	271	0.007	Antisense oligonucleotides ASOs	47	0.004
Binding	1575	0.009	Crystal structure	247	0.009	Peptide nucleic acids	44	0.007
Analysis	1548	0.009	Secondary structure	232	0.008	RNA interference RNAi	41	0.004
Molecular	1493	0.009	Antiparallel stretch	222	0.008	Non-coding RNAs lncRNAs	40	0.007
Expression	1383	0.008	Hydrogen bonds	201	0.009	Isothermal titration calorimetry	38	0.006
Data	1253	0.007	Circular dichroism	197	0.009	Transcription start sites	36	0.004
Antiparallel	1110	0.006	Binding sites	188	0.008	Single nucleotide polymorphisms	34	0.006
Cells	1044	0.006	Mayor groove	187	0.009	Triple helix formation	33	0.003
Proteins	988	0.006	DNA binding	168	0.008	Human immunodeficiency virus	33	0.004
Model	978	0.006	Modeling study	153	0.006	Northern blot analysis	33	0.004
Interactions	896	0.006	Dynamic simulations	148	0.006	Magnetic Resonance Spectroscopy	32	0.007
Oligonucleotides	777	0.004	Triple helix	130	0.006	Natural antisense transcripts	31	0.006

^a^ Unigrams: keywords composed of only one word. ^b^ Bigrams: keywords composed of two words. ^c^ Trigrams: keywords composed of three words.

## Data Availability

No new data were created or analyzed in this study.

## Supplementary Materials

The following supporting information can be downloaded at: https://www.mdpi.com/article/10.3390/ijms262210936/s1.
