# Supplementary material for: Antisense Versus Antigene in the Computer-Aided Design of Triplex-Forming Oligonucleotides (TFO): Insights from a Dual-Method Review, Combining Bibliometric and Systematic Review
Source: Int J Mol Sci. 2025 Nov 12;26(22):10936. doi: 10.3390/ijms262210936 (PMC12652294; doi:10.3390/ijms262210936)
Supplement: Supplementary file 1 [file ijms-26-10936-s001.zip › ijms-3913568-supplementary.pdf]

PRISMA 2020 Checklist

Antisense versus Antigene in the Computer-Aided Design of Triplex Forming Oligonucleotides (TFO): Insights from a Dual-Method Review, Combining Bibliometric and Systematic Review

Section and Topic	Item #	Checklist item	Location where item is reported
**TITLE**			
Title	1	Identify the report as a systematic review.	Lines 1 to 4
**ABSTRACT**			
Abstract	2	See the PRISMA 2020 for Abstracts checklist.	Line 30<br>Line 49
**INTRODUCTION**			
Rationale	3	Describe the rationale for the review in the context of existing knowledge.	Lines 68 to 78
Objectives	4	Provide an explicit statement of the objective(s) or question(s) the review addresses.	Lines 180 to 182<br>Lines 625 to 629
**METHODS**			
Eligibility criteria	5	Specify the inclusion and exclusion criteria for the review and how studies were grouped for the syntheses.	Lines 518 to 565<br>Lines 630 to 641
Information sources	6	Specify all databases, registers, websites, organisations, reference lists and other sources searched or consulted to identify studies. Specify the date when each source was last searched or consulted.	Lines 518 to 526<br>Lines 538 to 544<br>Lines 642 to 660
Search strategy	7	Present the full search strategies for all databases, registers and websites, including any filters and limits used.	Lines 518 to 537<br>Lines 550 to 565<br>Lines 642 to 660
Selection process	8	Specify the methods used to decide whether a study met the inclusion criteria of the review, including how many reviewers screened each record and each report retrieved, whether they worked independently, and if applicable, details of automation tools used in the process.	Lines 630 to 641<br>Lines 670 to 747
Data collection process	9	Specify the methods used to collect data from reports, including how many reviewers collected data from each report, whether they worked independently, any processes for obtaining or confirming data from study investigators, and if applicable, details of automation tools used in the process.	Lines 661 to 747
Data items	10a	List and define all outcomes for which data were sought. Specify whether all results that were compatible with each outcome domain in each study were sought (e.g. for all measures, time points, analyses), and if not, the methods used to decide which results to collect.	Lines 630 to 641<br>Lines 661 to 675
	10b	List and define all other variables for which data were sought (e.g. participant and intervention characteristics, funding sources). Describe any assumptions made about any missing or unclear information.	Lines 670 to 704
Study risk of bias assessment	11	Specify the methods used to assess risk of bias in the included studies, including details of the tool(s) used, how many reviewers assessed each study and whether they worked independently, and if applicable, details of automation tools used in the process.	Lines 685 to 693<br>Lines 793 to 794<br>Lines 1129 to 1130
Effect measures	12	Specify for each outcome the effect measure(s) (e.g. risk ratio, mean difference) used in the synthesis or presentation of results.	Lines 676 to 684

PRISMA 2020 Checklist

Antisense versus Antigene in the Computer-Aided Design of Triplex Forming Oligonucleotides (TFO): Insights from a Dual-Method Review, Combining Bibliometric and Systematic Review

Section and Topic	Item #	Checklist item	Location where item is reported
Synthesis methods	13a	Describe the processes used to decide which studies were eligible for each synthesis (e.g. tabulating the study intervention characteristics and comparing against the planned groups for each synthesis (item #5)).	Lines 630 to 669<br>Lines 676 to 694<br>Line 983<br>Lines 986 to 987<br>Lines 991 to 992<br>Lines 994 to 997
	13b	Describe any methods required to prepare the data for presentation or synthesis, such as handling of missing summary statistics, or data conversions.	Lines 670 to 675<br>Line 677<br>Lines 685 to 747
	13c	Describe any methods used to tabulate or visually display results of individual studies and syntheses.	Lines 676 to 684<br>Line 716<br>Lines 736 to 738
	13d	Describe any methods used to synthesize results and provide a rationale for the choice(s). If meta-analysis was performed, describe the model(s), method(s) to identify the presence and extent of statistical heterogeneity, and software package(s) used.	Lines 676 to 684<br>Lines 681 to 684<br>Lines 742 to 794<br>Lines 787 to 788<br>Lines 803 to 804<br>Lines 956 to 960<br>Lines 985 to 986<br>Lines 979 to 992<br>Line 1015<br>Lines 1056 to 1057<br>Lines 1132 to 1134
	13e	Describe any methods used to explore possible causes of heterogeneity among study results (e.g. subgroup analysis, meta-regression).	Lines 742 to 794<br>Lines 979 to 992<br>Lines 1056 to 1057<br>Lines 1132 to 1134
	13f	Describe any sensitivity analyses conducted to assess robustness of the synthesized results.	Lines 987 to 989<br>Lines 1015 to 1016<br>Lines 1057 to 1058<br>Lines 1132 to 1134

Antisense versus Antigene in the Computer-Aided Design of Triplex Forming Oligonucleotides (TFO): Insights from a Dual-Method Review, Combining Bibliometric and Systematic Review

Section and Topic	Item #	Checklist item	Location where item is reported
Reporting bias assessment	14	Describe any methods used to assess risk of bias due to missing results in a synthesis (arising from reporting biases).	Lines 685 to 693<br>Lines 793 to 794<br>Lines 969 to 972<br>Lines 1124 to 1134
Certainty assessment	15	Describe any methods used to assess certainty (or confidence) in the body of evidence for an outcome.	Lines 694 to 699<br>Line 1130
**RESULTS**			
Study selection	16a	Describe the results of the search and selection process, from the number of records identified in the search to the number of studies included in the review, ideally using a flow diagram.	Lines 642 to 660<br>Lines 705 to 747<br>Lines 752 to 794<br>Lines 949 to 968
	16b	Cite studies that might appear to meet the inclusion criteria, but which were excluded, and explain why they were excluded.	Lines 630 to 641<br>Lines 705 to 747<br>Lines 949 to 968
Study characteristics	17	Cite each included study and present its characteristics.	Lines 954 to 960<br>Line 998<br>Lines 973 to 1075<br>Lines 1014 to 1015<br>Lines 1055 to 1057
Risk of bias in studies	18	Present assessments of risk of bias for each included study.	Lines 793 to 794<br>Lines 969 to 972<br>Lines 1129 to 1130
Results of individual studies	19	For all outcomes, present, for each study: (a) summary statistics for each group (where appropriate) and (b) an effect estimate and its precision (e.g. confidence/credible interval), ideally using structured tables or plots.	Lines 973 to 1075
Results of syntheses	20a	For each synthesis, briefly summarise the characteristics and risk of bias among contributing studies.	Lines 982 to 984
	20b	Present results of all statistical syntheses conducted. If meta-analysis was done, present for each the summary estimate and its precision (e.g. confidence/credible interval) and measures of statistical heterogeneity. If comparing groups, describe the direction of the effect.	Lines 803 to 804<br>Lines 982 to 992
	20c	Present results of all investigations of possible causes of heterogeneity among study results.	Lines 979 to 981<br>Lines 982 to 983<br>Lines 986 to 992

Antisense versus Antigene in the Computer-Aided Design of Triplex Forming Oligonucleotides (TFO): Insights from a Dual-Method Review, Combining Bibliometric and Systematic Review

Section and Topic	Item #	Checklist item	Location where item is reported
			Lines 1132 to 1134
	20d	Present results of all sensitivity analyses conducted to assess the robustness of the synthesized results.	Lines 987 to 989<br>Lines 1132 to 1133
Reporting biases	21	Present assessments of risk of bias due to missing results (arising from reporting biases) for each synthesis assessed.	Lines 793 to 794<br>Lines 969 to 972<br>Lines 979 to 981<br>Lines 983 to 984<br>Lines 1129 to 1131
Certainty of evidence	22	Present assessments of certainty (or confidence) in the body of evidence for each outcome assessed.	Lines 993 to 997<br>Lines 1015 to 1016<br>Lines 1057 to 1058<br>Line 1130
**DISCUSSION**			
Discussion	23a	Provide a general interpretation of the results in the context of other evidence.	Lines 799 to 804<br>Lines 998 to 1008
	23b	Discuss any limitations of the evidence included in the review.	Lines 38 to 41<br>Line 998<br>Lines 1076 to 1104<br>Lines 1167 to 1171
	23c	Discuss any limitations of the review processes used.	Lines 676 to 704<br>Lines 690 to 693<br>Lines 743 to 794<br>Lines 803 to 804<br>Lines 969 to 972<br>Lines 979 to 998<br>Lines 1015 to 1016<br>Lines 1056 to 1058<br>Lines 1124 to 1134
	23d	Discuss implications of the results for practice, policy, and future research.	Line 998<br>Lines 1105 to 1123<br>Lines 1135 to 1160

PRISMA 2020 Checklist

Antisense versus Antigene in the Computer-Aided Design of Triplex Forming Oligonucleotides (TFO): Insights from a Dual-Method Review, Combining Bibliometric and Systematic Review

Section and Topic	Item #	Checklist item	Location where item is reported
**OTHER INFORMATION**			
Registration and protocol	24a	Provide registration information for the review, including register name and registration number, or state that the review was not registered.	Lines 700 to 704<br>Line 794<br>Lines 1131 to 1132
	24b	Indicate where the review protocol can be accessed, or state that a protocol was not prepared.	Lines 700 to 704<br>Line 794<br>Lines 1131 to 1132
	24c	Describe and explain any amendments to information provided at registration or in the protocol.	Lines 700 to 704<br>Line 794<br>Lines 1131 to 1132
Support	25	Describe sources of financial or non-financial support for the review, and the role of the funders or sponsors in the review.	Lines 1196 to 1201
Competing interests	26	Declare any competing interests of review authors.	Line 1205
Availability of data, code and other materials	27	Report which of the following are publicly available and where they can be found: template data collection forms; data extracted from included studies; data used for all analyses; analytic code; any other materials used in the review.	Lines 1203 to 1204

From: Page MJ, McKenzie JE, Bossuyt PM, Boutron I, Hoffmann TC, Mulrow CD, et al. The PRISMA 2020 statement: an updated guideline for reporting systematic reviews. BMJ 2021;372:n71. doi: 10.1136/bmj.n71.
This work is licensed under CC BY 4.0. To view a copy of this license, visit https://creativecommons.org/licenses/by/4.0/
